# The implication of chromosomal abnormalities in the surgical outcomes of Chinese pediatric patients with congenital heart disease

**DOI:** 10.3389/fcvm.2023.1164577

**Published:** 2023-05-24

**Authors:** Xiafeng Yu, Yu Tao, Xu Liu, Feng Yu, Chuan Jiang, Yingying Xiao, Haibo Zhang, Yongrui He, Lincai Ye, Ying Wang, Chunxia Zhou, Jian Wang, Zhengwen Jiang, Haifa Hong

**Affiliations:** ^1^ Department of Cardiothoracic Surgery, Shanghai Children’s Medical Center, Shanghai Jiaotong University School of Medicine, Shanghai, China; ^2^ Department of Genetics, Genesky Biotechnologies Inc., Shanghai, China; ^3^ Institute of Pediatric Congenital Heart Disease, Shanghai Children’s Medical Center, Shanghai Jiaotong University School of Medicine, Shanghai, China; ^4^ Department of Medical Genetics and Molecular Diagnostic Laboratory, Shanghai Children’s Medical Center, Shanghai Jiaotong University School of Medicine, Shanghai, China

**Keywords:** congenital heart disease, pediatric, copy number variation, ligation-dependent probe amplification, surgery

## Abstract

**Background:**

Copy number variations (CNVs) have been shown to be overrepresented in children with congenital heart disease (CHD). Genetic evaluation of CHD is currently underperformed in China. We sought to determine the occurrence of CNVs in CNV regions with disease-causing potential among a large cohort of Chinese pediatric CHD patients and investigate whether these CNVs could be the important critical modifiers of surgical intervention.

**Methods:**

CNVs screenings were performed in 1,762 Chinese children who underwent at least one cardiac surgery. CNV status at over 200 CNV locus with disease-causing potential was analyzed with a high-throughput ligation-dependent probe amplification (HLPA) assay.

**Results:**

We found 378 out of 1,762 samples (21.45%) to have at least one CNV and 2.38% of them were carrying multiple CNVs. The detection rates of ppCNVs (pathogenic and likely pathogenic CNVs) were 9.19% (162/1,762), significantly higher than that of the healthy Han Chinese individuals from The Database of Genomic Variants archive (9.19% vs. 3.63%; *P* = 0.0012). CHD cases with ppCNVs had a significantly higher proportion of complex surgeries compared to CHD patients with no ppCNVs (62.35% vs. 37.63%, *P* < 0.001). Duration of cardiopulmonary bypass and aortic cross clamp procedures were significantly longer in CHD cases with ppCNVs (all *P* < 0.05), while no group differences were identified for complications of surgery and one-month mortality after surgery. The detection rate of ppCNVs in the atrioventricular septal defect (AVSD) subgroup was significantly higher than that in other subgroups (23.10% vs. 9.70%, *P* = 0.002).

**Conclusions:**

CNV burden is an important contributor to Chinese children with CHD. Our study demonstrated the robustness and diagnostic efficiency of HLPA method in the genetic screening of CNVs in CHD patients.

## Introduction

Congenital heart disease (CHD), the most common type of birth-related structural and/or functional anomaly in the heart, affects nearly 1% of live births per year (1, 2). It has also been one of the leading causes of infant mortality, especially among children aged under 5 years in China (1). Although the rate of survival has been improved after the surgical intervention, the rate of long-term morbidity remains high. CHD encompasses a wide spectrum of congenital cardiac abnormalities, which greatly differ with respect to function, anatomy, and clinical outcomes (3).

The underlying mechanisms driving the gene mutation-associated CHD onset are complex and remain poorly understood, which include chromosomal aneuploidy (deletion or duplication of chromosomes), copy number variation (CNV), *de novo* mutation, and single-nucleotide polymorphism (SNP) (4, 5, 6, 7). Notably, any of these mechanisms can impact the function of certain gene products playing critical roles in healthy cardiac development and activity.

Approximately 12% of the human genome is believed to be regulated by CNV-mediated gene duplication or deletion mechanism (8), and pathogenic and/or large CNVs have been over-represented in a substantial proportion of CHD patients (9, 10). Subsequently, multiple recurrent CNV deletion loci, such as 22q11.2, 7q11.23, and 8p23.1, have been found to confer substantial risks for syndromic or isolated CHD (11, 12, 13). Additionally, large CNVs in CHD cases are associated with neurodevelopmental deficits, cognitive dysfunction, and poor clinical outcomes (14). For example, a genetic study of 422 cases with non-syndromic CHD found that the presence of pathogenic CNVs had a significantly negative impact on transplant-free survival and interventions (15). CNV-impacted diverse alterations in the chromosomal landscape have been extensively profiled by various genetic studies, such as fluorescence *in situ* hybridization (FISH) (16), array-based comparative genomic hybridization (aCGH) (17), single nucleotide polymorphism array (18), and next-generation sequencing (NGS) (19). However, most of the published reports included cohorts with limited sample sizes, and large-scale screening of CHD-related CNV was not performed in Chinese CHD patients. The high-throughput ligation-dependent probe amplification (HLPA) assay is an alternative CNV-screening tool with a great cost-benefit, minimum turn-around time, and high compatibility with multiplex PCR systems (20). HLPA has also shown high concordance with the aCGH-based CNV detection with respect to cost, time, and precision (21). Furthermore, the HLPA method has also been applied to identify the pathogenic CNV, 15q11.2 deletion, in a rare form of CHD, called total anomalous pulmonary venous connection (TAPVC) (22).

Thus, we sought to assess the frequency of occurrence of CNVs in over 200 recurrent CNV locus with potential pathogenic effect among a large cohort of Chinese pediatric patients with CHD using HLPA assay and investigate whether these CNVs could be the important critical modifiers of surgical intervention. We present the following article in accordance with the STROBE reporting checklist.

## Methods

### Study subjects and phenotype classification

A total of 1,762 patients (51% male, median age = 24.95 ± 30.78 months), who underwent at least one cardiac operation at Shanghai Children's Medical Center between January 2016 and January 2021, were recruited to this study. Past medical records, including admission notes and echocardiography (ECG) reports, were carefully reviewed during enrollment. This group of patients was evaluated using ECG, magnetic resonance imaging (MRI), cardiac catheterization, and/or surgical reports to diagnose specific CHD sub-type. Patients having only mild CHD abnormalities, such as isolated patent ductus arteriosus (PDA) and patent foramen ovale (PFO) or with a recognizable phenotypic syndrome, were excluded from this study.

### CNV detection by HLPA methodology

**Figure 1:**
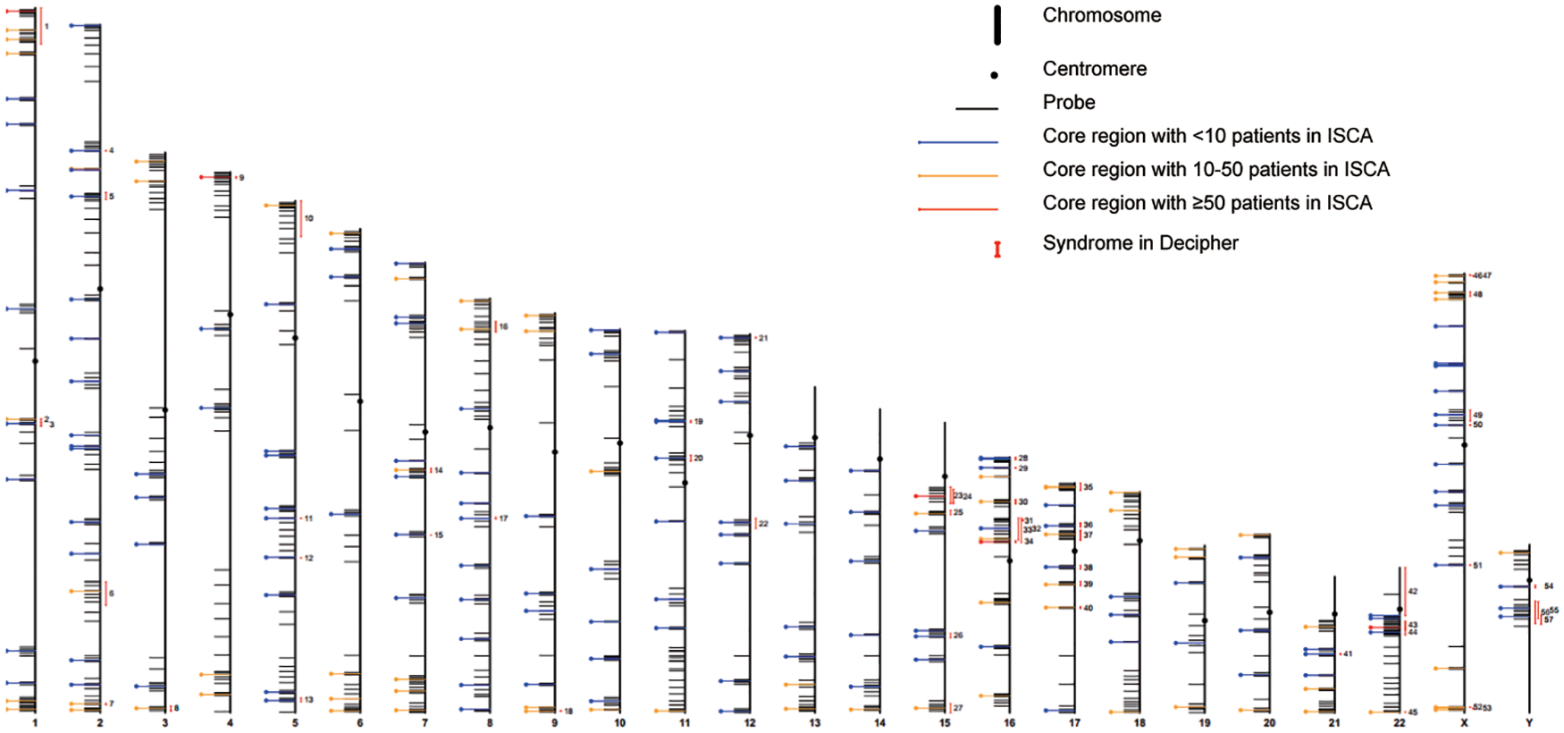
Genome-wide distribution of 1,602 probes on human chromosomes for CNV detection in CHD.

Genomic DNA was isolated from the 2 ml peripheral blood samples, collected from each subject during admission, using QIAamp DNA Blood kit (Qiagen, Hilden, Germany), following the manufacturer's procedures. CNV detection was performed with HLPA methodology. (D04T1006, Genesky, Suzhou, China),.HLPA is a modified version of the multiplex LPA (MLPA) method for the quantification of gene copy numbers in a multiplex PCR setup (23, 25). The validity and reliability of this method can be found elsewhere. Based on an earlier HLPA assay that used 170 and 341 pairs of probes to target each of the 24 chromosomes for the detection of aneuploidies (20, 23, 24)., the technique was enhanced by the addition of several hundred of pairs of probes to achieve a higher resolution for the detection of CNVs. This led to the inclusion of 1602 pairs of probes in the current assay to identify aneuploidies and CNVs. Briefly, a mixture of amplified fragments with various amplicon lengths, subsequently labeled with fluorophores, was prepared, which was then subjected to capillary electrophoresis for size-based separation and quantification of each labelled-amplicon. Peaks were retrieved with Genemapper V5.0 (Applied biosystems) and data analysis was performed by CNV Reader 1.0 (Genesky, Suzhou, China) (25). The relative value for each probe was then compared with the matching values obtained in all reference samples (inter-sample normalization). The final probe ratio was around 2.0 if the region of interest was unaffected by CNV, whereas an increased or decreased value indicated the duplication or deletion, respectively. Like MLPA, each probe contained a specific sequence complementary to the genomic target sequence as well as an adapter sequence that enabled exponential amplification of the template DNA using universal primers. HLPA method exploited a “lengthening” ligation system using a ligation template and a pair of elongated ligation probes to further increase the length of the downstream probe, further enabling the size-based simultaneous sorting of amplification products (20). MLPA, on the other hand, requires a stuffer sequence in the downstream probe that can reach a length of 200 bp, making it costly and challenging to chemically synthesis. Each lengthening probe had a sequence complementary to the ligation template and was hybridized in immediate proximity to each other. After hybridization, the probes specific to genomic DNA and the ligation template were ligated simultaneously by a thermally stable DNA ligase. The universal PCR primers were used to subsequently amplify the single/double ligated probes to obtain a contiguous DNA fragment. To further enhance the throughput, we used four types of 5′ universal primers labeled with different fluorophores and two types of 3′ primers that corresponded to the conventional 3′ probe and the lengthening ligation probe to amplify the ligated probes. A total of 1,602 probes were designed to pick up over 200 known recurrent CNV loci (Figure 1, Supplementary Table S1), including 200 core CNV regions and a range of regions flanking the core regions in the mitotic chromosome, genomic regions at about 0 Mb, 10 Mb, and 20 Mb distances from the telomeric end, and 3 gene-mutation loci (RBM8A: c.67 + 32G > C, c.−21delG; FGFR3: c.1138G > A), allowing the identification of CNV regions corresponding to 58 syndromes of CHD defined by DECIPHER (Supplementary Table S2). These regions were covered by at least 4 CNV segments and related to high-risk predisposition to congenital phylogenetic abnormalities as well as intellectual disability, according to the International Standards for Cytogenomic Array (ISCA) database of chromosomal aneuploidy, duplication, and deletion of the telomeric ends.

### Definition of CNV categories

Each CNV was classified into one of the following categories: (1) benign, (2) likely benign, (3) variants of uncertain significance, (4) likely pathogenic, or (5) pathogenic. Benign CNVs were defined as CNVs: (i) with an incidence of >1% in the Database of Genomic Variants (DGV); (ii) reported as benign in the ISCA or DECIFER database, and/or (iii) listed in the DGV with incidence below 1%, and do not contain any genes. Likely benign CNVs were defined as gene-containing variants detected multiple times in normal populations with <1% incidence rate. Variants of uncertain significance were defined as CNVs which were: (i) not eligible for the definition of pathogenic/likely pathogenic CNV nor benign CNV, (ii) not reported in the literature, and/or (iii) lacking sufficient evidence to make a definitive classification. Likely pathogenic CNVs were defined as deletions or duplications that: (i) partially overlapped with reported pathogenic deletions or duplications; (ii) affected genes that were suspected, but not functionally confirmed, in the pathogenesis of the disease, and/or (iii) affected genes for which there was evidence to support double- or triple-dose sensitivity, but not enough to draw firm conclusions. Pathogenic CNVs were defined as deletions or duplications that matched in location and size to the previously identified pathogenic regions in established microdeletion/microduplication syndromes. Likely pathogenic and pathogenic CNVs were grouped together as “potentially pathogenic” CNVs (ppCNVs).

### Statistical analysis

SPSS 20.0 software was used for statistical analysis. Student t-test was applied for the continuous variables, while Chi-squared (*χ*^2^) test or the Fisher's exact test was applied to compare groups whenever appropriate. A *P*-value of <0.05 was considered statistically significant.

## Results

### Diagnostic yield of CNV testing for children with CHD

Between January 2016 and January 2021, about 17,708 patients received CHD correction surgeries at Shanghai Children's Medical Center and a total of 1,762 samples were eligible for the CNV analysis. Among the subjects, 51.0% were male with a mean age of 24.95 ± 30.78 months. Out of these patients, 703 individuals (39.90%) underwent complex surgeries and 159 (9.02%) individuals had multiple surgeries.

**Figure 2:**
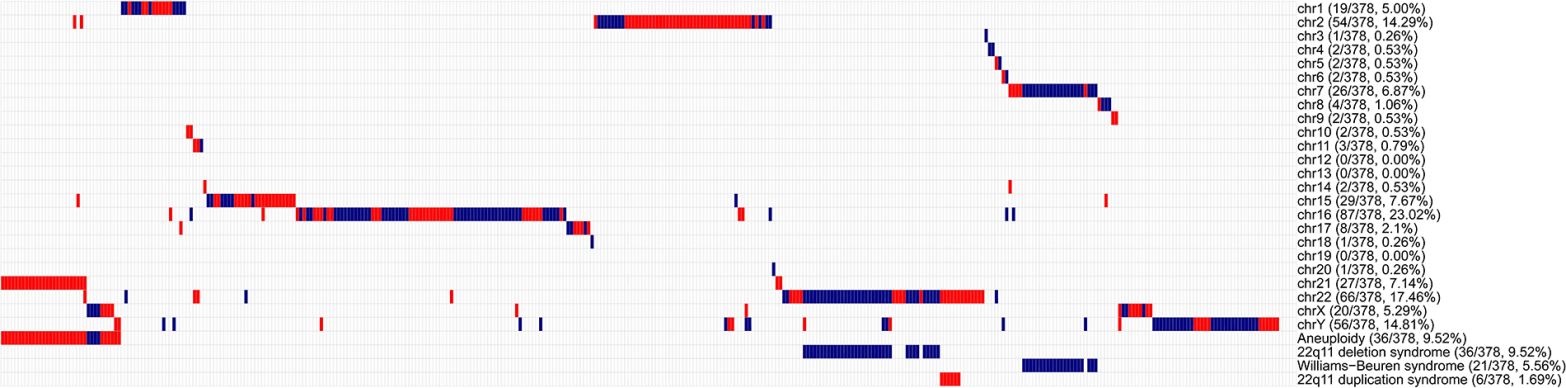
Overview of chromosomal aberrations detected in 378 patients by the HLPA assay A plot shows the CNV status of each of the 378 individuals on each chromosome, as labeled on the right. Each column represents a sample. Duplications are depicted by red bars, and deletions are depicted by blue bars. High proportions of CNVs are found in genomic regions correlated with aneuploidy, 22q11 deletion syndrome, Williams-Beuren syndrome, and 22q11 duplication syndrome, as labeled on the right.

The overall diagnostic yield of CNV testing for children with CHD was 21.45% (378/1,762), while 2.38% (42/1,762) of these patients carried multiple CNV. A summary of chromosomal abnormalities in 378 CHD patients is provided in Table 1 and Figure 2, and detailed information is provided in Supplementary Table S3. Clinically, ppCNV abnormalities were detected in 162 of 1,762 samples (9.19%), including 36 cases with whole chromosome aneuploidies (2.04%), 42 cases with 22q11 deletions or duplications (2.38%), and 84 cases with microdeletions or duplications (4.77%) related to other known chromosomal disease syndromes.

**Table 1:** Summary of chromosomal abnormalities of CHD patients.

CNV carriers (*N* = 378^b^)	Type	CNVs category	Details	
	Aneuploidy (*N* = 36)	Pathogenic^a^	Triploid (*N* = 32)	47, + 21 (*N* = 25)
				47, XXX (*N* = 4)
				46, XY/47, XYY chimeras (*N* = 1)
				47, XXY (*N* = 2)
			Monosomies	45, X0 (*N* = 4)
	Syndromes related micro-deletion and -duplication (*N* = 120)	Pathogenic^a^	22q11 deletion syndrome (*N* = 36)	
			Williams-Beuren syndrome (*N* = 21)	
			22q11 duplication syndrome (*N* = 6)	
			16p13.11 microdeletions or duplications syndromes (neurocognitive disorder susceptibility locus) (*N* = 4)	
			15q11.2 microdeletion syndrome (*N* = 4)	
			1q21.1 susceptibility locus for Thrombocytopenia-Absent Radius (TAR) syndrome (*N* = 4)	
			1q21.1 microduplication (*N* = 4)	
			Charcot-Marie-Tooth syndrome (*N* = 2)	
			22q11.2 distal deletion syndrome (1)	
			3q29 microdeletion syndrome (*N* = 1)	
			2p21 microdeletion syndrome (*N* = 1)	
			Miller-Dieker syndrome (*N* = 1)	
			15q13.3 microdeletion syndrome (*N* = 1)	
			15q24 microdeletion syndrome (*N* = 1)	
			16p12.1 microdeletion syndrome (*N* = 1)	
			Renal cysts and diabetic syndrome (*N* = 1)	
			Leri-Weill chondrogenesis disorder syndrome (*N* = 1)	
			Cri du Chat syndrome (*N* = 1)	
			8p23.1 microdeletion syndrome (*N* = 1)	
			7q11.23 duplication syndrome (*N* = 1)	
			Hereditary neuropathy with liability to pressure palsy syndrome (*N* = 1)	
	Other micro-deletion and -duplication	Pathogenic^a^	Chr1:243782460–246739575 deletion(*N* = 1)	
			Chr8:413101–6302778 deletion (*N* = 1)	
			Chr9:130742496–141008509 duplication (*N* = 1)	
			Chr1:864351–4529446 deletion (*N* = 1)	
			Chr7:139121221–159029030 duplication (*N* = 1)	
			Chr8:413101–6302778 deletion (*N* = 1)	
			Chr15:72923127–101887270 duplication (*N* = 1)	
		Likely pathogenic^a^	(*N* = 26)	
			A detailed list can be found in Supplementary Table S3.	
		Benign	(*N* = 103)	
			A detailed list can be found in Supplementary Table S3.	
		Likely benign	(*N* = 68)	
			HBA duplication (27)	
			2q13(NPHP1) microduplication (39)	
			HBA deletion (*N* = 43)	
		Variants of uncertain significance	(*N* = 33)	
			A detailed list can be found in Supplementary Table S3.	

^a^ CNVs belonging to these categories were defined as potentially pathogenic CNVs.

^b^ 2.38% (42/1,762) of CHD patients were carrying multiple CNVs.

**Figure 3:**
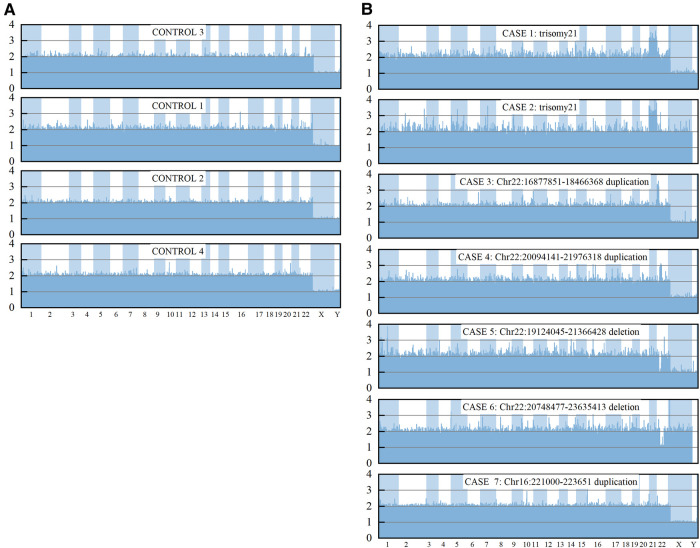
CNV measurements for control samples and a subset of CHD by HLPA. The final probe ratio depicted in the Y axis was around 2.0 if the region of interest was unaffected by CNV, whereas an increased or decreased value indicated the duplication or deletion, respectively.

In comparison, 11 individuals with 4 such potentially pathogenic CNVs were identified in 303 healthy Han Chinese individuals from DGV database (http://dgv.tcag.ca/dgv/docs/GRCh37_hg19_variants_2020-02-25.txt), according to 90% reciprocal overlap criterion (Supplementary Table S4). Significant difference in the proportion of ppCNV events between the CHD cases and the controls (9.19% vs. 3.63%; *P* = 0.0012) was presented. Results from a subset of CHD cases and three control individuals showed that a reliable 24 chromosome copy number profile could be obtained using the HLPA assay (Figure 3).

Baseline characteristics and clinical data related to the first surgery of the CHD cases stratified by presence or absence of CNV or ppCNVs are presented in Supplementary Table S5 and Table 2. CHD cases with CNV exhibited a significantly higher proportion of complex surgeries (48.68% vs. 37.50%, *P* < 0.001). Duration of surgery time for cardiopulmonary bypass (CPBT), aortic cross clamp (ACCT) and mechanical ventilation procedures (MVT) were significantly longer in CHD cases with CNV (all *P* < 0.05). There were no significant differences in terms of age, weight and gender distribution between the two groups. No significant differences were identified between the groups for incidences of delayed sternal closure, continuous positive airway pressure, open-chest hemostasis, use of extracorporeal membrane oxygenation (ECMO) or left ventricular assist device (LVAD) systems, complications of surgery or one-month mortality after surgery.

**Table 2:** Baseline characteristics of CHD patients (stratified by the presence or absence of ppCNVs).

Characteristics	potentially pathogenic CNV present	potentially pathogenic CNV absent	Combined	*P*-value
Sample size	162	1,600	1,762	
Male, %	81 (50.0%)	817 (51.1%)	898 (51.96%)	0.797
Female, %	81 (50.0%)	783 (48.9%)	864 (48.04%)	
Age, months	24.35 ± 30.38	25.02 ± 30.83	24.95 ± 30.78	0.792
Weight, kg	10.35 ± 5.78	11.50 ± 7.75	11.39 ± 7.60	**0** **.** **021**
Complex surgery, %				
YES	101 (62.35%)	602 (37.63%)	703 (39.90%)	**<0**.**001**
NO	61 (37.65%)	998 (62.37%)	1,058 (60.10%)	
CPBT, min	77.62 ± 53.58	62.61 ± 42.50	63.99 ± 43.84	**0**.**001**
ACCT, min	42.30 ± 29.57	33.19 ± 26.11	33.99 ± 26.58	**<0**.**001**
MVT, min	80.56 ± 192.32	53.95 ± 149.07	56.38 ± 153.66	**0**.**090**
Delayed sternal closure, %				
YES	5 (3.09%)	42 (2.63%)	47 (2.67%)	0.728
NO	157 (96.91%)	1,558 (97.37%)	1,715 (97.33%)	** **
Hemostasis, %				
YES	1 (0.62%)	8 (0.50%)	9 (0.51%)	0.842
NO	161 (99.38%)	1,592 (99.50%)	1,753 (99.49%)	
ECMO or LVAD, %				
YES	1 (0.62%)	11 (0.69%)	12 (0.68%)	0.918
NO	161 (99.38%)	1,589 (99.31%)	1,750 (99.32%)	
Diaphragmatic paralysis, %				
YES	2 (1.23%)	13 (0.81%)	15 (0.85%)	0.577
NO	160 (98.77%)	1,587 (99.19%)	1,747 (99.15%)	
Hypoxemia, %				
YES	9 (5.56%)	60 (3.75%)	69 (3.92%)	0.259
NO	153 (94.44%)	1,540 (96.25%)	1,693 (96.08%)	
Neurological complications, %				
YES	0 (0.0%)	2 (0.13%)	2 (0.11%)	0.653
NO	162 (100%)	1,598 (99.87%)	1,760 (99.89%)	
HF, %				
YES	5 (3.09%)	32 (2.00%)	37 (2.10%)	0.358
NO	157 (96.91%)	1,568 (98.00%)	1,725 (97.90%)	** **
MOF, %				
YES	2 (1.23%)	21 (1.31%)	23 (1.31%)	0.934
NO	160 (98.77%)	1,579 (98.69%)	1,739 (98.69%)	
RF, %				
YES	6 (3.70%)	32 (2.00%)	38 (2.16%)	0.155
NO	156 (96.30%)	1,568 (98.00%)	1,724 (97.84%)	
Infection, %				
YES	6 (3.70%)	29 (1.81%)	39 (2.21%)	0.100
NO	156 (96.30%)	1,571 (98.19%)	1,723 (97.79%)	** **
Outcome, %				
Cured	160 (98.77%)	1,591 (99.44%)	1,751 (99.38%)	0.433
one-month mortality after surgery	2 (1.23%)	9 (0.56%)	11 (0.62%)	

Complex surgery: surgeries for CHD subtypes with the exception of the atrial septal defect, ventricular septal defect, patent ductus arteriosus, and isolated pulmonic stenosis; CPBT, cardiopulmonary bypass time; ACCT, aortic cross-clamp time; MVT, mechanical ventilation time; ECMO, extracorporeal membrane oxygenation; LVAD, left ventricular assist device; HF, hepatic failure; MOF, multiple organ failure; RF, renal failure.

In total, 162 CHD cases were identified with ppCNVs and had significantly higher incidences of complex surgeries compared to that of non-carriers (62.35% vs. 37.63%, *P* < 0.001). Durations for cardiopulmonary bypass and aortic cross clamp procedures were significantly longer in CHD cases with ppCNVs (all *P* < 0.05). There were no significant differences with respect to age, gender distribution or ventilation times between the two groups. No significant differences were identified between the groups for incidences of delayed sternal closure, continuous positive airway pressure, open-chest hemostasis, use of ECMO or LVAD systems, surgical complications, and one-month mortality after surgery.

### Sub-group analysis of CHD sub-types

**Figure 4:**
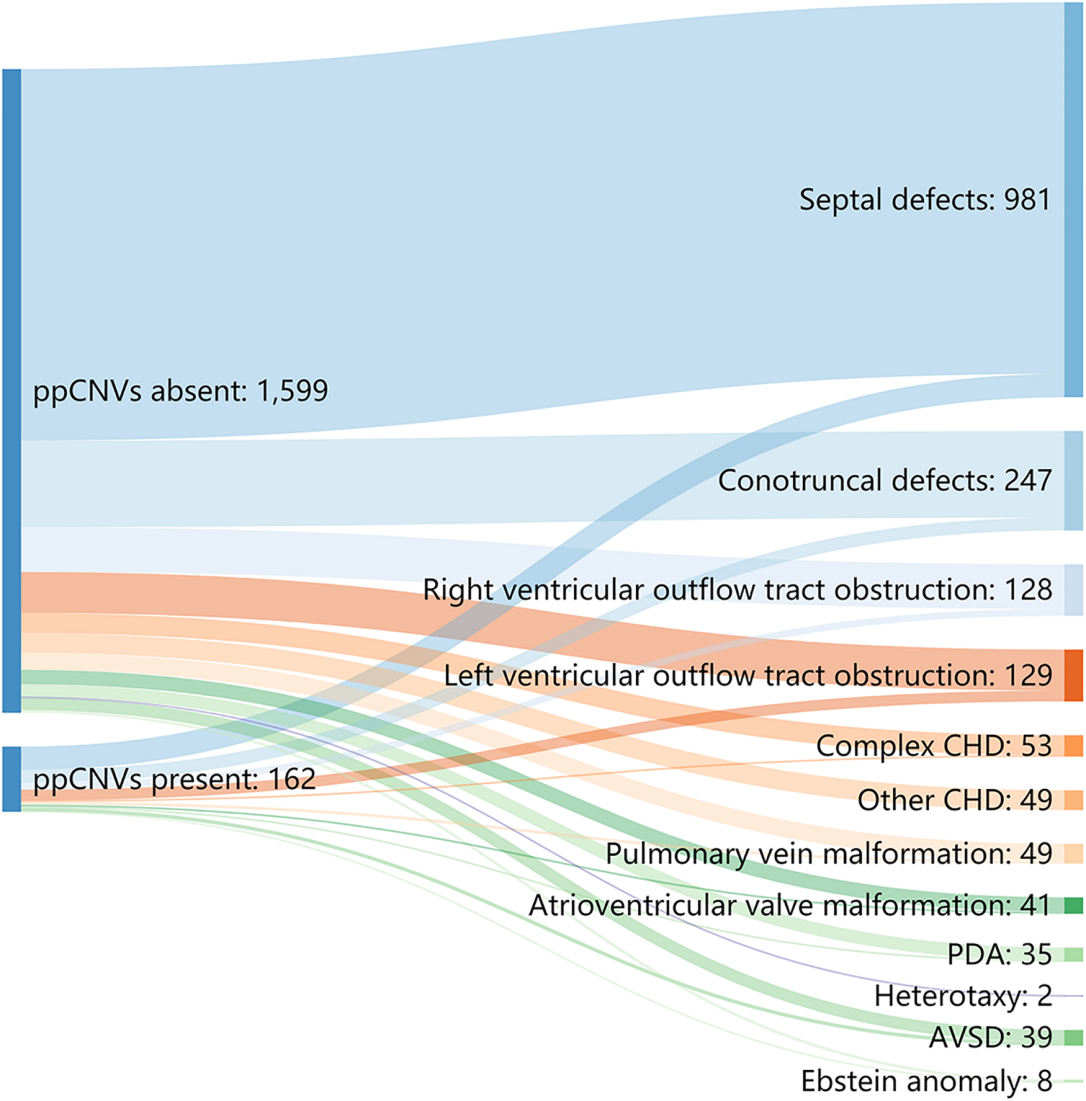
Sankey diagram describing the relative flow of CHD sub-groups according to the presence or absence of ppCNVs. CHD, congenital heart disease; PDA, patent ductus arteriosus; AVSD, atrioventricular septal defect.

We categorized all children with CHD into 12 sub-groups. Detection number and rates of ppCNV findings in different CHD sub-groups are listed in Table 3 and Figure 4. Overall, the septal defect, defined as ventricular septal defect and atrial septal defect were the most observed heart malformation affecting 981 cases (55.67%), followed by conotruncal defects in 247 cases (14.02%). Patients with atrioventricular septal defect (AVSD) (23.10%) and left ventricular outflow tract obstruction (LVOTO) (20.90%) were diagnosed with high detection rates of ppCNV abnormalities, while patients with other CHD (0.00%) and AVSD (5.90%) symptoms exhibited low detection rates. The detection rate of patients with ppCNVs in the AVSD sub-group was significantly higher than that in the other sub-groups (23.10% vs. 9.70%, *P* = 0.002).

**Table 3:** Rates of chromosomal abnormalities in different CHD sub-groups.

Subgroups		ppCNVs			
		absent	present	*N*	*P*-value
Heterotaxy	*N*	2	0	2	0.653
	% in each subgroup	100.00%	0%		
Ebstein anomaly	*N*	7	1	8	0.747
	% in each subgroup	87.50%	12.50%		
Patent ductus arteriosus	*N*	32	3	35	0.899
	% in each subgroup	91.40%	8.60%		
Atrioventricular septal defect	*N*	30	9	39	**0** **.** **003**
	% in each subgroup	76.90%	23.10%		
Atrioventricular valve malformation	*N*	36	5	41	0.512
	% in each subgroup	87.80%	12.20%		
Pulmonary vein malformation	*N*	42	7	49	0.227
	% in each subgroup	85.70%	14.30%		
Other CHD	*N*	49	0	49	**0** **.** **026**
	% in each subgroup	100.0%	0%		
Complex CHD	*N*	50	3	53	0.378
	% in each subgroup	94.30%	5.70%		
Left ventricular outflow tract obstruction	*N*	102	27	129	**<0** **.** **001**
	% in each subgroup	79.10%	20.90%		
Right ventricular outflow tract obstruction	*N*	111	17	128	0.127
	% in each subgroup	86.70%	13.30%		
Conotruncal defects	*N*	215	32	247	0.061
	% in each subgroup	87.00%	13.00%		
Septal defects	*N*	923	58	981	**0** **.** **002**
	% in each subgroup	94.10%	5.90%		
TOTAL	*N*	1,600	162	1,762	
	%	90.80%	9.20%	100.0%	

Complex CHD was defined as functional single ventricle and malformation of coronary artery; Conotruncal defects were defined as a group of congenital heart defects with abnormal ventriculo-arterial connections.

## Discussion

Genome-wide association studies of pathogenic and/or rare CNVs have highlighted the significant contribution of genetic variations toward CHD susceptibility (26, 27, 28, 29). In this study, 1,762 CHD patients, who underwent corrective surgeries for cardiac defects, were genetically screened for rare CNVs. The overall detection rate of ppCNVs was 9.19%, indicating a substantial genetic heterogeneity among the patients and the detection rate was slightly lower in accordance with previous studies (19, 30).

Notably, 2.04% of the CNV anomalies were aneuploidies, of which trisomy 21 was the most common chromosomal anomaly in these patients. Since most babies with Down syndrome present CHD, early screening and surgical interventions can greatly increase their survival rate and the life quality (31). Multiple deletion and duplication syndromes have been described both clinically and mechanistically (32). In many cases, genetic analysis of these syndromes has provided insights into the etiology of associated congenital anomalies. For example, the recognition that the majority of patients with DiGeorge syndrome carry a 22q11.2 deletion led to the discovery that a large number of cardiac patients with a subset of syndromic phenotypes might have 22q11.2 deletion (33). Further molecular studies have demonstrated that the T-Box transcription factor 1 (*TBX1*) gene*,* located within the 22q11.2 region, plays a critical role in the development of the abnormal cardiac phenotypes (34). Thus, we hypothesized that screening for known CNVs associated with the aberrant chromosomal disease might facilitate the genetic diagnosis of CHD in pediatric patients. Interestingly, we found 126 individuals with CNVs implicated in the known microdeletion or duplication syndromes, including 42 individuals with 22q11 microdeletions or duplications (2.38%) and 87 (4.77%) related to other chromosomal disease syndromes. Since the clinical manifestations of certain chromosomal diseases syndromes occur later than cardiac abnormalities, for example, abnormal neurodevelopmental phenotypes, genetic screening for CHD patients is highly recommended.

While CNV burden has previously been reported to be an important modifier of survival outcomes following CHD surgeries (15), our study did not identify correlation between CNV status and one-month survival after surgery. Further exploration with longer follow-up time is warranted. Our study was the first to investigate the contribution of CNVs to the complexity of corrective surgeries for cardiac defects. As described earlier, CHD cases with ppCNVs had a significantly higher proportion of complex surgeries. Time of turnover and blocking were also significantly longer in CHD cases with ppCNVs. Considering the long-term and complex impact of CNV burden, CHD patients should be genetically screened to evaluate the necessity of correction surgeries prior to surgical interventions for better disease management (35). Further research in this area is highly recommended to improve the treatment plan.

Consistent with the findings by Wang et al. (30), the septal defect was the most common type of heart malformation in our cohort, followed by conotruncal defects, with similar detection rates. Although Wang et al. demonstrated that the detection rate of pathogenic chromosomal abnormalities in fetuses with AVSD was significantly higher than those in the other sub-groups, the rate of incidence in these study sub-groups was lower- 73.7% in Wang et al. vs. 23.10% in this study. Furthermore, LVOTO (20.90%) was revealed to harbor a higher rate of ppCNV abnormalities compared with other studies (13.30% (19) and 0% (30)). These differences in rates could be due to differences in sample sizes and/or the proportions of isolated CHD cases between these studies.

Several high-throughput techniques, including SNP arrays, CGH arrays, and NGS-based assays are now widely used in genome-wide association studies (GWAS) to investigate the broader implications of chromosomal abnormalities in human diseases. These techniques, however, are expensive and, thus, may not be suitable for the preliminary rapid screenings in the clinics. Alternatively, other techniques, including FISH and MLPA, have also been used in recent studies. Despite FISH being the gold standard method for detecting chromosomal abnormalities, the technique has some key limitations, such as the time-consuming, laborious, requirement for experienced personnel and low-throughput efficiency, preventing its routine use in diagnostics. MLPA, a clinically effective targeted test, provides easily interpretable results also has a constraint of probe number (36). On the other hand, HLPA shares several advantages of MLPA, including high target specificity, since DNA probes require perfect complementarity to the target sequences for efficient ligation. Additionally, HLPA employs optimized lengthening ligation steps, allowing the use of a large number of short probes compared to MLPA. Notably, the use of short probes increases both annealing and amplification efficiencies, thereby, reducing the assay variability. In this study, we demonstrated that HLPA method could detect more than 200 genomic loci in a single reaction, using probes separated with an average chromosomal distance of 10 Mb, suggesting that CNVs with the sizes larger than 10 Mb can be readily detected. The turnaround time was within 24 h and, importantly, the cost for HLPA was substantially lower than that of other techniques (e.g., 1/10 of the cost of aCGH). Thus, the HLPA assay method was found to be the most suitable one with respect to higher genome coverage, accuracy, and cost-effectiveness.

Some limitations of this study should be considered. First, by concentrating on the overall burden of identified ppCNVs in healthy Han Chinese people from public datasets, we were only able to address the rarity of each particular CNV, and statistical power was constrained due to the absence of comparable cohorts. Second, due to the limited number of genomic samples of parents of the affected CHD cases, we were not able to determine whether the ppCNVs reported in our study were *de novo* or inherited. Third, like MLPA and aCGH techniques, which depend upon comparisons with reference chromosomes to determine the copy number, HLPA was unable to detect polyploidies. Moreover, due to the limited screening resolution, it could not detect small size microdeletions/duplications or complex structural rearrangements, such as balanced translocations and inversions. Therefore, the detection rates in this study might underestimate the actual rate of incidence of chromosome abnormalities. Finally, as the prevalence of individuals living with CHD has been increasing due to advances in pediatric surgery, more attention is needed concerning the contributions of CNV abnormalities on long-term surgical outcomes. We are currently conducting a 5-year follow-up study to evaluate the impact of CNVs on patients' outcomes.

In summary, this study focuses on the CHD pathology in children provides significant insights into the genomic landscape of Chinese CHD patients. Our data suggest that CNV burden could be an important contributor to the Chinese pediatric CHD patients. Our study also demonstrated the robustness of HLPA method for profiling CNVs in CHD patients, indicating the clinical potential of this technique toward its routine application in the clinical setting prior to cardiac surgery.

## Data Availability

The original contributions presented in the study are included in the article/**Supplementary Materials**, further inquiries can be directed to the corresponding author/s.
