# Supplementary material for: The implication of chromosomal abnormalities in the surgical outcomes of Chinese pediatric patients with congenital heart disease
Source: Front Cardiovasc Med. 2023 May 24;10:1164577. doi: 10.3389/fcvm.2023.1164577 (PMC10244782; doi:10.3389/fcvm.2023.1164577)
Supplement: Supplementary file 1 [file Datasheet1.pdf]

Supplementary Table S1.Psysical location of 1,602 probes on the human genome in HLPA assay.

Probe Number	Probe location (hg19)	Probe Number	Probe location (hg19)	Probe Number	Probe location (hg19)	Probe Number	Probe location (hg19)
1	Chr1:864351	401	Chr5:109655958	801	Chr11:47282179	1201	Chr17:44270208
2	Chr1:959928	402	Chr5:112062927	802	Chr11:50174937	1202	Chr17:61082356
3	Chr1:1087297	403	Chr5:112095932	803	Chr11:55170782	1203	Chr17:71151312
4	Chr1:1169768	404	Chr5:112121485	804	Chr11:67051710	1204	Chr17:77295249
5	Chr1:1271000	405	Chr5:112157612	805	Chr11:67132173	1205	Chr17:77884701
6	Chr1:1333630	406	Chr5:113603736	806	Chr11:67226286	1206	Chr17:78789338
7	Chr1:1860774	407	Chr5:116304460	807	Chr11:67325755	1207	Chr17:79222215
8	Chr1:2762529	408	Chr5:118556213	808	Chr11:67430538	1208	Chr17:80038422
9	Chr1:3098743	409	Chr5:121400892	809	Chr11:91083025	1209	Chr17:80244040
10	Chr1:3761882	410	Chr5:123589894	810	Chr11:93029350	1210	Chr17:80390329
11	Chr1:4529446	411	Chr5:125819195	811	Chr11:94903357	1211	Chr17:81044340
12	Chr1:7913532	412	Chr5:125929501	812	Chr11:96123395	1212	Chr17:81152056
13	Chr1:7972138	413	Chr5:126113717	813	Chr11:97134423	1213	Chr18:158979
14	Chr1:8037244	414	Chr5:126162742	814	Chr11:104741477	1214	Chr18:180306
15	Chr1:8114738	415	Chr5:138208886	815	Chr11:104931374	1215	Chr18:225806
16	Chr1:8916545	416	Chr5:138678266	816	Chr11:105019222	1216	Chr18:251424
17	Chr1:9370023	417	Chr5:139292758	817	Chr11:105169234	1217	Chr18:274207
18	Chr1:10511480	418	Chr5:139303458	818	Chr11:105396875	1218	Chr18:346896
19	Chr1:11140851	419	Chr5:139418928	819	Chr11:111974734	1219	Chr18:732572
20	Chr1:11186801	420	Chr5:139511608	820	Chr11:113102911	1220	Chr18:1146158
21	Chr1:11217308	421	Chr5:139820621	821	Chr11:113331129	1221	Chr18:1219735
22	Chr1:11254082	422	Chr5:145205595	822	Chr11:114941279	1222	Chr18:1707570
23	Chr1:11288818	423	Chr5:149631607	823	Chr11:115520943	1223	Chr18:2892253
24	Chr1:11709437	424	Chr5:161494741	824	Chr11:116619000	1224	Chr18:3153134
25	Chr1:12202860	425	Chr5:164513885	825	Chr11:116871596	1225	Chr18:3496579
26	Chr1:15639660	426	Chr5:166319367	826	Chr11:119521789	1226	Chr18:4125380
27	Chr1:16043232	427	Chr5:168180975	827	Chr11:120186119	1227	Chr18:5144374
28	Chr1:16199371	428	Chr5:170235703	828	Chr11:123476067	1228	Chr18:6522032
29	Chr1:16330752	429	Chr5:172860328	829	Chr11:126079482	1229	Chr18:7909857
30	Chr1:16641863	430	Chr5:173383129	830	Chr11:128329336	1230	Chr18:9400481
31	Chr1:31819534	431	Chr5:173566994	831	Chr11:130848715	1231	Chr18:12155503
32	Chr1:32257935	432	Chr5:173705932	832	Chr11:131865332	1232	Chr18:13387739
33	Chr1:32541317	433	Chr5:174130369	833	Chr11:132204694	1233	Chr18:18545686

34	Chr1:32834084	434	Chr5:175813609	834	Chr11:132285190	1234	Chr18:35857953
35	Chr1:40997289	435	Chr5:175976920	835	Chr11:133565060	1235	Chr18:36382415
36	Chr1:41159022	436	Chr5:176165224	836	Chr11:133710574	1236	Chr18:37066888
37	Chr1:41242004	437	Chr5:176562744	837	Chr11:134322741	1237	Chr18:37662211
38	Chr1:41326978	438	Chr5:176665323	838	Chr11:134359731	1238	Chr18:38417425
39	Chr1:41750895	439	Chr5:176700732	839	Chr11:134394894	1239	Chr18:41869856
40	Chr1:62971437	440	Chr5:176946057	840	Chr11:134401471	1240	Chr18:43211045
41	Chr1:64563483	441	Chr5:177026142	841	Chr11:134444189	1241	Chr18:43422451
42	Chr1:64609176	442	Chr5:178747095	842	Chr11:134604993	1242	Chr18:43619975
43	Chr1:64696199	443	Chr5:180239219	843	Chr12:432791	1243	Chr18:44085943
44	Chr1:67450532	444	Chr5:180698263	844	Chr12:701132	1244	Chr18:52981461
45	Chr1:104868160	445	Chr6:483508	845	Chr12:706804	1245	Chr18:53007271
46	Chr1:105524599	446	Chr6:931416	846	Chr12:1137399	1246	Chr18:53089500
47	Chr1:106497848	447	Chr6:1439269	847	Chr12:1354137	1247	Chr18:53140451
48	Chr1:107045985	448	Chr6:1448891	848	Chr12:1604941	1248	Chr18:53191969
49	Chr1:107866925	449	Chr6:1604426	849	Chr12:1604941	1249	Chr18:63205444
50	Chr1:120469916	450	Chr6:1612782	850	Chr12:1991596	1250	Chr18:65179946
51	Chr1:145438533	451	Chr6:1614882	851	Chr12:2258020	1251	Chr18:66344384
52	Chr1:145507403	452	Chr6:1726673	852	Chr12:2684281	1252	Chr18:67957972
53	Chr1:145513361	453	Chr6:1850414	853	Chr12:3347768	1253	Chr18:70410742
54	Chr1:145556660	454	Chr6:2198404	854	Chr12:3649893	1254	Chr18:72997515
55	Chr1:145630244	455	Chr6:2514017	855	Chr12:4303841	1255	Chr18:73921090
56	Chr1:145696018	456	Chr6:3113645	856	Chr12:6437350	1256	Chr18:74178992
57	Chr1:145747058	457	Chr6:3281772	857	Chr12:11804029	1257	Chr18:74695817
58	Chr1:146627532	458	Chr6:4426708	858	Chr12:11907359	1258	Chr18:75055121
59	Chr1:146696520	459	Chr6:6266890	859	Chr12:12829088	1259	Chr18:75540647
60	Chr1:146806886	460	Chr6:6770299	860	Chr12:13120763	1260	Chr18:75852135
61	Chr1:146942359	461	Chr6:7187709	861	Chr12:13410026	1261	Chr18:76162492
62	Chr1:147083442	462	Chr6:7611745	862	Chr12:14881036	1262	Chr18:77005314
63	Chr1:147120096	463	Chr6:8240226	863	Chr12:15773836	1263	Chr18:77733416
64	Chr1:147213036	464	Chr6:16300025	864	Chr12:20799788	1264	Chr18:77806536
65	Chr1:147293834	465	Chr6:16761649	865	Chr12:21931806	1265	Chr18:77868274
66	Chr1:150082629	466	Chr6:16843327	866	Chr12:23811472	1266	Chr18:77915512
67	Chr1:153974327	467	Chr6:17036080	867	Chr12:23854685	1267	Chr18:77981694
68	Chr1:165322351	468	Chr6:17093386	868	Chr12:23923138	1268	Chr19:1014228
69	Chr1:166732629	469	Chr6:17401884	869	Chr12:24390779	1269	Chr19:1194514

70	Chr1:166786801	470	Chr6:17409891	870	Chr12:34754917	1270	Chr19:1207185
71	Chr1:166845605	471	Chr6:20100285	871	Chr12:38641034	1271	Chr19:1230433
72	Chr1:167218284	472	Chr6:20204650	872	Chr12:65626710	1272	Chr19:1271192
73	Chr1:226055654	473	Chr6:25554303	873	Chr12:66221825	1273	Chr19:2241749
74	Chr1:226751026	474	Chr6:58612863	874	Chr12:66747219	1274	Chr19:3347279
75	Chr1:227181097	475	Chr6:71321936	875	Chr12:67507105	1275	Chr19:3586503
76	Chr1:227602307	476	Chr6:99382686	876	Chr12:70523597	1276	Chr19:4046298
77	Chr1:228338800	477	Chr6:100160445	877	Chr12:70788071	1277	Chr19:4075356
78	Chr1:229210512	478	Chr6:100843284	878	Chr12:70907020	1278	Chr19:4078670
79	Chr1:238535934	479	Chr6:100882088	879	Chr12:70954576	1279	Chr19:4095112
80	Chr1:238577052	480	Chr6:100911362	880	Chr12:71314340	1280	Chr19:4268950
81	Chr1:238698502	481	Chr6:100927224	881	Chr12:80829496	1281	Chr19:4422452
82	Chr1:238816996	482	Chr6:101489064	882	Chr12:80936575	1282	Chr19:4498850
83	Chr1:239360034	483	Chr6:108372259	883	Chr12:81013186	1283	Chr19:4748169
84	Chr1:243782488	484	Chr6:112386078	884	Chr12:81101607	1284	Chr19:13033316
85	Chr1:244114046	485	Chr6:117710546	885	Chr12:81294994	1285	Chr19:13084536
86	Chr1:244232186	486	Chr6:150863843	886	Chr12:113812381	1286	Chr19:13184254
87	Chr1:244780955	487	Chr6:156839603	887	Chr12:122079862	1287	Chr19:13232172
88	Chr1:245028398	488	Chr6:157287148	888	Chr12:122332586	1288	Chr19:13345754
89	Chr1:245159890	489	Chr6:157338808	889	Chr12:122618451	1289	Chr19:13424870
90	Chr1:245346896	490	Chr6:157410453	890	Chr12:122962438	1290	Chr19:24229119
91	Chr1:245636730	491	Chr6:157710836	891	Chr12:123814308	1291	Chr19:28265676
92	Chr1:245775063	492	Chr6:161139344	892	Chr12:129706707	1292	Chr19:31765937
93	Chr1:245777832	493	Chr6:162740421	893	Chr12:130525724	1293	Chr19:33597632
94	Chr1:246613736	494	Chr6:164455949	894	Chr12:131295374	1294	Chr19:35447703
95	Chr1:246625688	495	Chr6:166198348	895	Chr12:132308531	1295	Chr19:37854761
96	Chr1:246739597	496	Chr6:166890815	896	Chr12:132418026	1296	Chr19:49095088
97	Chr1:247095487	497	Chr6:167491212	897	Chr12:132863889	1297	Chr19:53819869
98	Chr1:247293676	498	Chr6:168136406	898	Chr12:133197610	1298	Chr19:56019945
99	Chr1:247491211	499	Chr6:168662648	899	Chr12:133336793	1299	Chr19:56499180
100	Chr1:248051451	500	Chr6:168990193	900	Chr12:133481385	1300	Chr19:56985923
101	Chr1:248260994	501	Chr6:169204319	901	Chr12:133816936	1301	Chr19:57120583
102	Chr1:248556490	502	Chr6:169614952	902	Chr13:19809466	1302	Chr19:57339919
103	Chr2:39137	503	Chr6:169857542	903	Chr13:20665603	1303	Chr19:57481318
104	Chr2:230015	504	Chr6:170166955	904	Chr13:20823844	1304	Chr19:57946750
105	Chr2:339697	505	Chr6:170428629	905	Chr13:20978151	1305	Chr19:58205022

106	Chr2:495058	506	Chr6:170575722	906	Chr13:21071188	1306	Chr19:59096741
107	Chr2:855415	507	Chr6:170742397	907	Chr13:21635929	1307	Chr20:80206
108	Chr2:1227267	508	Chr7:143462	908	Chr13:21966860	1308	Chr20:123286
109	Chr2:1997953	509	Chr7:339149	909	Chr13:29506669	1309	Chr20:199697
110	Chr2:2067279	510	Chr7:638242	910	Chr13:32162251	1310	Chr20:244830
111	Chr2:2781934	511	Chr7:1070955	911	Chr13:32605554	1311	Chr20:307565
112	Chr2:3191912	512	Chr7:1562754	912	Chr13:32977210	1312	Chr20:594161
113	Chr2:3655623	513	Chr7:1678213	913	Chr13:33130166	1313	Chr20:825356
114	Chr2:4662746	514	Chr7:2394551	914	Chr13:33334797	1314	Chr20:1147128
115	Chr2:7179792	515	Chr7:2941413	915	Chr13:45801523	1315	Chr20:1210691
116	Chr2:10271362	516	Chr7:3224063	916	Chr13:47691944	1316	Chr20:1699197
117	Chr2:14774121	517	Chr7:4875367	917	Chr13:48348618	1317	Chr20:4483987
118	Chr2:20035978	518	Chr7:5232115	918	Chr13:48868069	1318	Chr20:4702036
119	Chr2:41366921	519	Chr7:5460951	919	Chr13:51418060	1319	Chr20:7222342
120	Chr2:42020121	520	Chr7:5618973	920	Chr13:83183154	1320	Chr20:7549262
121	Chr2:42950078	521	Chr7:5692097	921	Chr13:84522109	1321	Chr20:8113032
122	Chr2:43575849	522	Chr7:5732508	922	Chr13:84764340	1322	Chr20:8582697
123	Chr2:44396679	523	Chr7:5792379	923	Chr13:85007746	1323	Chr20:10864871
124	Chr2:44413048	524	Chr7:5835580	924	Chr13:85669506	1324	Chr20:13491595
125	Chr2:44503208	525	Chr7:18720322	925	Chr13:94375590	1325	Chr20:14318308
126	Chr2:44508655	526	Chr7:19202016	926	Chr13:95055642	1326	Chr20:16828416
127	Chr2:44545813	527	Chr7:19325659	927	Chr13:95071042	1327	Chr20:25732660
128	Chr2:44553227	528	Chr7:19443040	928	Chr13:95583999	1328	Chr20:29844956
129	Chr2:44575731	529	Chr7:19629288	929	Chr13:96233007	1329	Chr20:33293180
130	Chr2:50826887	530	Chr7:20795650	930	Chr13:97085468	1330	Chr20:33703272
131	Chr2:50848032	531	Chr7:21200861	931	Chr13:103607833	1331	Chr20:33859506
132	Chr2:50855311	532	Chr7:21781565	932	Chr13:105177877	1332	Chr20:33981591
133	Chr2:50860616	533	Chr7:22581082	933	Chr13:106095530	1333	Chr20:34430572
134	Chr2:50868515	534	Chr7:23383925	934	Chr13:109459066	1334	Chr20:42909670
135	Chr2:50890479	535	Chr7:24486698	935	Chr13:111009836	1335	Chr20:48787490
136	Chr2:50914975	536	Chr7:26452488	936	Chr13:111318071	1336	Chr20:49455564
137	Chr2:50947080	537	Chr7:57318870	937	Chr13:111866158	1337	Chr20:49626852
138	Chr2:51113515	538	Chr7:62509709	938	Chr13:112290197	1338	Chr20:49735839
139	Chr2:51173597	539	Chr7:69947708	939	Chr13:112611408	1339	Chr20:50012037
140	Chr2:51195324	540	Chr7:69970248	940	Chr13:112970553	1340	Chr20:58301590
141	Chr2:51206575	541	Chr7:70063625	941	Chr13:113153180	1341	Chr20:59372856

142	Chr2:51217961	542	Chr7:70203062	942	Chr13:113615994	1342	Chr20:60454001
143	Chr2:51231108	543	Chr7:72858332	943	Chr13:113990987	1343	Chr20:60965512
144	Chr2:51256096	544	Chr7:73113653	944	Chr13:114322197	1344	Chr20:61432325
145	Chr2:51275303	545	Chr7:73262134	945	Chr13:114544398	1345	Chr20:62078721
146	Chr2:51294880	546	Chr7:73483745	946	Chr14:21574828	1346	Chr20:62095964
147	Chr2:51435811	547	Chr7:74020657	947	Chr14:21697808	1347	Chr20:62120116
148	Chr2:59617996	548	Chr7:74071238	948	Chr14:21795802	1348	Chr20:62149633
149	Chr2:59971219	549	Chr7:75185353	949	Chr14:21883907	1349	Chr20:62376724
150	Chr2:60386245	550	Chr7:75517955	950	Chr14:21996936	1350	Chr20:62643073
151	Chr2:60995936	551	Chr7:75877538	951	Chr14:30335026	1351	Chr20:62821621
152	Chr2:61708396	552	Chr7:75998446	952	Chr14:36036483	1352	Chr21:15480245
153	Chr2:62280838	553	Chr7:95125351	953	Chr14:36169397	1353	Chr21:15843945
154	Chr2:64860731	554	Chr7:95727328	954	Chr14:36340654	1354	Chr21:16906081
155	Chr2:68765257	555	Chr7:96005621	955	Chr14:36480536	1355	Chr21:17476158
156	Chr2:73482374	556	Chr7:96318654	956	Chr14:36789702	1356	Chr21:17943403
157	Chr2:80529365	557	Chr7:96339188	957	Chr14:37126936	1357	Chr21:18296070
158	Chr2:84957351	558	Chr7:96970960	958	Chr14:41093020	1358	Chr21:19207993
159	Chr2:95626946	559	Chr7:117307340	959	Chr14:52118684	1359	Chr21:25400485
160	Chr2:96754344	560	Chr7:117824121	960	Chr14:52999608	1360	Chr21:25484188
161	Chr2:97130701	561	Chr7:118550136	961	Chr14:53589068	1361	Chr21:25669698
162	Chr2:97529813	562	Chr7:119295677	962	Chr14:53905585	1362	Chr21:25740263
163	Chr2:110873515	563	Chr7:120388147	963	Chr14:54417337	1363	Chr21:25816350
164	Chr2:110907779	564	Chr7:139121246	964	Chr14:79560190	1364	Chr21:26408347
165	Chr2:110932096	565	Chr7:146139697	965	Chr14:79791325	1365	Chr21:27253940
166	Chr2:110954953	566	Chr7:146739311	966	Chr14:79825037	1366	Chr21:27375017
167	Chr2:123135406	567	Chr7:147003845	967	Chr14:79861465	1367	Chr21:27447791
168	Chr2:124783244	568	Chr7:147600719	968	Chr14:79905477	1368	Chr21:27479135
169	Chr2:126049123	569	Chr7:148357742	969	Chr14:87254166	1369	Chr21:28089880
170	Chr2:127267326	570	Chr7:148954324	970	Chr14:95679620	1370	Chr21:34616481
171	Chr2:128520497	571	Chr7:150171278	971	Chr14:96722565	1371	Chr21:34788017
172	Chr2:144108987	572	Chr7:151064140	972	Chr14:97619796	1372	Chr21:34926483
173	Chr2:145021990	573	Chr7:151411093	973	Chr14:98542923	1373	Chr21:35107330
174	Chr2:145086428	574	Chr7:151818031	974	Chr14:99927427	1374	Chr21:39427029
175	Chr2:145201395	575	Chr7:152160336	975	Chr14:101405215	1375	Chr21:39670129
176	Chr2:148778595	576	Chr7:154445433	976	Chr14:103083697	1376	Chr21:39897618
177	Chr2:148934910	577	Chr7:155715274	977	Chr14:104030642	1377	Chr21:40219177

178	Chr2:148944552	578	Chr7:156931974	978	Chr14:104897976	1378	Chr21:43778716
179	Chr2:148953589	579	Chr7:157049562	979	Chr14:105146255	1379	Chr21:44180522
180	Chr2:149031446	580	Chr7:157646174	980	Chr14:105375763	1380	Chr21:44586310
181	Chr2:149216018	581	Chr7:157952057	981	Chr14:105643881	1381	Chr21:44969918
182	Chr2:149829872	582	Chr7:157996511	982	Chr14:106033995	1382	Chr21:45848763
183	Chr2:150171116	583	Chr7:158141249	983	Chr14:106153421	1383	Chr21:46795349
184	Chr2:151903393	584	Chr7:158394785	984	Chr14:106252752	1384	Chr21:46954128
185	Chr2:155308938	585	Chr7:158734785	985	Chr14:106398097	1385	Chr21:47033732
186	Chr2:157181712	586	Chr7:159001084	986	Chr14:106509848	1386	Chr21:47278604
187	Chr2:174633864	587	Chr7:159029051	987	Chr15:22848894	1387	Chr21:47609620
188	Chr2:174938910	588	Chr8:413123	988	Chr15:22868862	1388	Chr21:47817582
189	Chr2:175711771	589	Chr8:751406	989	Chr15:22923707	1389	Chr21:47865516
190	Chr2:176126823	590	Chr8:962955	990	Chr15:22929790	1390	Chr21:48089327
191	Chr2:176679977	591	Chr8:1223394	991	Chr15:23000108	1391	Chr22:16868912
192	Chr2:183387537	592	Chr8:1478949	992	Chr15:23006798	1392	Chr22:16877851
193	Chr2:186896884	593	Chr8:2089786	993	Chr15:23021203	1393	Chr22:16945442
194	Chr2:186966317	594	Chr8:2129614	994	Chr15:23049242	1394	Chr22:17305131
195	Chr2:187031466	595	Chr8:3216655	995	Chr15:23052195	1395	Chr22:17447361
196	Chr2:189156517	596	Chr8:3425309	996	Chr15:23300241	1396	Chr22:17466066
197	Chr2:196709850	597	Chr8:4555989	997	Chr15:23889303	1397	Chr22:17770290
198	Chr2:197122595	598	Chr8:6302798	998	Chr15:24924384	1398	Chr22:18031589
199	Chr2:198262728	599	Chr8:8247146	999	Chr15:25931143	1399	Chr22:18243546
200	Chr2:200173585	600	Chr8:8994014	1000	Chr15:26726257	1400	Chr22:18466368
201	Chr2:200246966	601	Chr8:9953182	1001	Chr15:27918075	1401	Chr22:19124045
202	Chr2:200255498	602	Chr8:10782180	1002	Chr15:31324152	1402	Chr22:19745145
203	Chr2:200265695	603	Chr8:11617732	1003	Chr15:31460705	1403	Chr22:19758119
204	Chr2:200298134	604	Chr8:11654857	1004	Chr15:31570617	1404	Chr22:19766670
205	Chr2:201342701	605	Chr8:11696306	1005	Chr15:31726085	1405	Chr22:20094141
206	Chr2:202485656	606	Chr8:14273363	1006	Chr15:31851209	1406	Chr22:20748477
207	Chr2:204031995	607	Chr8:16184311	1007	Chr15:32066410	1407	Chr22:21098965
208	Chr2:207651589	608	Chr8:22029199	1008	Chr15:32149059	1408	Chr22:21153695
209	Chr2:210794664	609	Chr8:26513469	1009	Chr15:32271170	1409	Chr22:21212916
210	Chr2:223034383	610	Chr8:32405819	1010	Chr15:32376199	1410	Chr22:21269834
211	Chr2:223807890	611	Chr8:36629507	1011	Chr15:32410688	1411	Chr22:21335519
212	Chr2:224549187	612	Chr8:38268703	1012	Chr15:32440462	1412	Chr22:21366428
213	Chr2:224647930	613	Chr8:38934873	1013	Chr15:36872021	1413	Chr22:21411131

214	Chr2:224840132	614	Chr8:39792703	1014	Chr15:37420670	1414	Chr22:21976318
215	Chr2:225148149	615	Chr8:41549087	1015	Chr15:38243574	1415	Chr22:22182761
216	Chr2:231050511	616	Chr8:42704832	1016	Chr15:38765764	1416	Chr22:22342545
217	Chr2:232839877	617	Chr8:53135174	1017	Chr15:39267505	1417	Chr22:22518649
218	Chr2:233103295	618	Chr8:61433274	1018	Chr15:72923148	1418	Chr22:22763807
219	Chr2:233386282	619	Chr8:61535340	1019	Chr15:73263699	1419	Chr22:22839259
220	Chr2:234681517	620	Chr8:61571135	1020	Chr15:73654035	1420	Chr22:23022064
221	Chr2:237246871	621	Chr8:61879432	1021	Chr15:74144059	1421	Chr22:23210459
222	Chr2:238831721	622	Chr8:72124123	1022	Chr15:74627405	1422	Chr22:23315504
223	Chr2:239260625	623	Chr8:72191743	1023	Chr15:75337249	1423	Chr22:23420970
224	Chr2:239922215	624	Chr8:72268721	1024	Chr15:75645012	1424	Chr22:23511274
225	Chr2:239938876	625	Chr8:72314436	1025	Chr15:75706601	1425	Chr22:23635413
226	Chr2:239969937	626	Chr8:72467807	1026	Chr15:82379884	1426	Chr22:24106301
227	Chr2:240153839	627	Chr8:75227322	1027	Chr15:83350031	1427	Chr22:28144807
228	Chr2:240264600	628	Chr8:76291621	1028	Chr15:83638811	1428	Chr22:31328393
229	Chr2:241171940	629	Chr8:77616303	1029	Chr15:83925027	1429	Chr22:39003455
230	Chr2:241570510	630	Chr8:78565672	1030	Chr15:84541312	1430	Chr22:40859426
231	Chr2:241653236	631	Chr8:79717195	1031	Chr15:97684486	1431	Chr22:42767269
232	Chr2:241979515	632	Chr8:80578127	1032	Chr15:98374438	1432	Chr22:44892383
233	Chr2:242194495	633	Chr8:93848679	1033	Chr15:99496038	1433	Chr22:46770952
234	Chr2:242219857	634	Chr8:94053283	1034	Chr15:99564906	1434	Chr22:47791021
235	Chr2:242257684	635	Chr8:94279767	1035	Chr15:99721603	1435	Chr22:47990832
236	Chr2:242316651	636	Chr8:94508021	1036	Chr15:100015912	1436	Chr22:48462608
237	Chr2:242502139	637	Chr8:95205253	1037	Chr15:100642847	1437	Chr22:49301696
238	Chr2:242608257	638	Chr8:104831519	1038	Chr15:100739557	1438	Chr22:49576717
239	Chr3:672950	639	Chr8:106066028	1039	Chr15:101191576	1439	Chr22:50629603
240	Chr3:1443198	640	Chr8:106365712	1040	Chr15:101887293	1440	Chr22:50832453
241	Chr3:2142284	641	Chr8:106670389	1041	Chr16:218937	1441	Chr22:50929408
242	Chr3:2573122	642	Chr8:107858590	1042	Chr16:221000	1442	Chr22:51061350
243	Chr3:2874378	643	Chr8:118188072	1043	Chr16:221984	1443	Chr22:51127207
244	Chr3:3044277	644	Chr8:119941146	1044	Chr16:223618	1444	Chr22:51140601
245	Chr3:3103966	645	Chr8:120257118	1045	Chr16:225125	1445	Chr22:51148760
246	Chr3:3192285	646	Chr8:120596231	1046	Chr16:225336	1446	Chr22:51154319
247	Chr3:3222644	647	Chr8:120963664	1047	Chr16:227727	1447	ChrX:591793
248	Chr3:3301260	648	Chr8:126290780	1048	Chr16:495590	1448	ChrX:595387
249	Chr3:3886128	649	Chr8:131331118	1049	Chr16:596000	1449	ChrX:601750

250	Chr3:4536172	650	Chr8:133722393	1050	Chr16:694655	1450	ChrX:682028
251	Chr3:4557034	651	Chr8:136292646	1051	Chr16:796837	1451	ChrX:811412
252	Chr3:5163991	652	Chr8:136554991	1052	Chr16:1134959	1452	ChrX:817317
253	Chr3:6258038	653	Chr8:138200475	1053	Chr16:1406728	1453	ChrX:823169
254	Chr3:10018679	654	Chr8:139821416	1054	Chr16:1488963	1454	ChrX:838581
255	Chr3:10047402	655	Chr8:140629916	1055	Chr16:1715112	1455	ChrX:846121
256	Chr3:10099715	656	Chr8:142564764	1056	Chr16:2199642	1456	ChrX:2138488
257	Chr3:10145902	657	Chr8:143160969	1057	Chr16:3006488	1457	ChrX:2931105
258	Chr3:10212140	658	Chr8:144622812	1058	Chr16:3623491	1458	ChrX:3012160
259	Chr3:10397365	659	Chr8:145086695	1059	Chr16:3704477	1459	ChrX:3120541
260	Chr3:11583051	660	Chr8:145138387	1060	Chr16:3731125	1460	ChrX:3163858
261	Chr3:12196441	661	Chr8:145181513	1061	Chr16:3742142	1461	ChrX:3248532
262	Chr3:14178902	662	Chr8:145502980	1062	Chr16:3752237	1462	ChrX:3345163
263	Chr3:16254102	663	Chr8:145757540	1063	Chr16:3777164	1463	ChrX:4042177
264	Chr3:17782847	664	Chr8:145776404	1064	Chr16:3852707	1464	ChrX:4999128
265	Chr3:20106049	665	Chr9:470059	1065	Chr16:3904595	1465	ChrX:5712221
266	Chr3:90195626	666	Chr9:586268	1066	Chr16:3985380	1466	ChrX:6456266
267	Chr3:93577172	667	Chr9:708625	1067	Chr16:4406882	1467	ChrX:6724171
268	Chr3:101038543	668	Chr9:968673	1068	Chr16:6800143	1468	ChrX:6757542
269	Chr3:105621035	669	Chr9:1318395	1069	Chr16:6858551	1469	ChrX:6764572
270	Chr3:109435712	670	Chr9:2077685	1070	Chr16:6940972	1470	ChrX:6774690
271	Chr3:112335586	671	Chr9:2095608	1071	Chr16:7017384	1471	ChrX:7151863
272	Chr3:113060702	672	Chr9:3023443	1072	Chr16:15057845	1472	ChrX:7368846
273	Chr3:113658813	673	Chr9:3219942	1073	Chr16:15166744	1473	ChrX:7698947
274	Chr3:114342984	674	Chr9:4231875	1074	Chr16:15507549	1474	ChrX:7867830
275	Chr3:114870898	675	Chr9:5079471	1075	Chr16:15623658	1475	ChrX:8530429
276	Chr3:121448798	676	Chr9:5570045	1076	Chr16:15644170	1476	ChrX:9035850
277	Chr3:121647866	677	Chr9:6328468	1077	Chr16:15741131	1477	ChrX:9159968
278	Chr3:121903301	678	Chr9:7169936	1078	Chr16:15847296	1478	ChrX:9275174
279	Chr3:122143527	679	Chr9:8872924	1079	Chr16:15911518	1479	ChrX:18669459
280	Chr3:122808120	680	Chr9:10405258	1080	Chr16:16169644	1480	ChrX:18676961
281	Chr3:137893591	681	Chr9:11318574	1081	Chr16:16235219	1481	ChrX:18683269
282	Chr3:138117365	682	Chr9:16595439	1082	Chr16:17406955	1482	ChrX:18700435
283	Chr3:138410096	683	Chr9:38771995	1083	Chr16:21601064	1483	ChrX:18764060
284	Chr3:138478160	684	Chr9:71074982	1084	Chr16:21995536	1484	ChrX:31763428
285	Chr3:138609739	685	Chr9:71131173	1085	Chr16:22105492	1485	ChrX:31788017

286	Chr3:178543470	686	Chr9:71391199	1086	Chr16:22256022	1486	ChrX:31812472
287	Chr3:182554193	687	Chr9:71667407	1087	Chr16:22826398	1487	ChrX:31850353
288	Chr3:187388175	688	Chr9:71944321	1088	Chr16:24046759	1488	ChrX:32242870
289	Chr3:187495292	689	Chr9:75504045	1089	Chr16:25189487	1489	ChrX:32299042
290	Chr3:188136855	690	Chr9:98638058	1090	Chr16:26147193	1490	ChrX:32325102
291	Chr3:188643067	691	Chr9:98857384	1091	Chr16:27180925	1491	ChrX:32356895
292	Chr3:189320472	692	Chr9:99071351	1092	Chr16:27214897	1492	ChrX:32680635
293	Chr3:190262400	693	Chr9:99277854	1093	Chr16:28846080	1493	ChrX:32721156
294	Chr3:194818696	694	Chr9:99636472	1094	Chr16:28875587	1494	ChrX:32761873
295	Chr3:195812808	695	Chr9:102018824	1095	Chr16:28916072	1495	ChrX:41402012
296	Chr3:196230197	696	Chr9:103421216	1096	Chr16:28943395	1496	ChrX:41494208
297	Chr3:196657816	697	Chr9:105188321	1097	Chr16:29008410	1497	ChrX:41586861
298	Chr3:196680303	698	Chr9:106436668	1098	Chr16:29674343	1498	ChrX:41714623
299	Chr3:197207589	699	Chr9:108152120	1099	Chr16:29703068	1499	ChrX:48316975
300	Chr3:197299909	700	Chr9:121008500	1100	Chr16:29791610	1500	ChrX:48979109
301	Chr4:101847	701	Chr9:130742496	1101	Chr16:29891333	1501	ChrX:49957019
302	Chr4:279474	702	Chr9:131051051	1102	Chr16:29938210	1502	ChrX:51079173
303	Chr4:629525	703	Chr9:131185450	1103	Chr16:29974874	1503	ChrX:52001188
304	Chr4:1318763	704	Chr9:131331081	1104	Chr16:30035837	1504	ChrX:53432015
305	Chr4:1513183	705	Chr9:131566382	1105	Chr16:30096147	1505	ChrX:53559945
306	Chr4:1617792	706	Chr9:136914059	1106	Chr16:30157283	1506	ChrX:53585696
307	Chr4:1718541	707	Chr9:138850263	1107	Chr16:33923074	1507	ChrX:53611282
308	Chr4:1875190	708	Chr9:139241701	1108	Chr16:35146897	1508	ChrX:53642742
309	Chr4:1932395	709	Chr9:139300600	1109	Chr16:48180462	1509	ChrX:53707035
310	Chr4:1957898	710	Chr9:139345740	1110	Chr16:50014265	1510	ChrX:53846681
311	Chr4:2407447	711	Chr9:139372116	1111	Chr16:50391564	1511	ChrX:58081199
312	Chr4:2513435	712	Chr9:139432620	1112	Chr16:50828305	1512	ChrX:66960993
313	Chr4:3204184	713	Chr9:139979250	1113	Chr16:51254191	1513	ChrX:67283894
314	Chr4:3485013	714	Chr9:140113461	1114	Chr16:51581927	1514	ChrX:67412789
315	Chr4:4297769	715	Chr9:140415318	1115	Chr16:51702363	1515	ChrX:67515836
316	Chr4:4386647	716	Chr9:140519681	1116	Chr16:52060323	1516	ChrX:76746917
317	Chr4:6711391	717	Chr9:140657213	1117	Chr16:66583395	1517	ChrX:76759162
318	Chr4:8318104	718	Chr9:140720036	1118	Chr16:66755784	1518	ChrX:76777475
319	Chr4:12031902	719	Chr9:140785173	1119	Chr16:67040865	1519	ChrX:76952514
320	Chr4:13542572	720	Chr9:140909362	1120	Chr16:67380236	1520	ChrX:77086897
321	Chr4:15937632	721	Chr9:141008509	1121	Chr16:67681225	1521	ChrX:77112232

322	Chr4:49083673	722	Chr10:147973	1122	Chr16:69976426	1522	ChrX:77141827
323	Chr4:53956293	723	Chr10:230943	1123	Chr16:83711853	1523	ChrX:77194006
324	Chr4:55095284	724	Chr10:267162	1124	Chr16:83842065	1524	ChrX:77253628
325	Chr4:55427033	725	Chr10:321116	1125	Chr16:84088155	1525	ChrX:77284153
326	Chr4:55808588	726	Chr10:818941	1126	Chr16:84362777	1526	ChrX:81022054
327	Chr4:57796076	727	Chr10:823970	1127	Chr16:84695698	1527	ChrX:81547002
328	Chr4:76841099	728	Chr10:1247860	1128	Chr16:85953549	1528	ChrX:82322929
329	Chr4:81974693	729	Chr10:2379326	1129	Chr16:86406828	1529	ChrX:83116177
330	Chr4:82510532	730	Chr10:2514049	1130	Chr16:86828245	1530	ChrX:84344544
331	Chr4:83218261	731	Chr10:3924005	1131	Chr16:86996447	1531	ChrX:94251769
332	Chr4:83261181	732	Chr10:4173102	1132	Chr16:87981912	1532	ChrX:96854843
333	Chr4:83520802	733	Chr10:7602444	1133	Chr16:88483850	1533	ChrX:99883457
334	Chr4:83745733	734	Chr10:8253506	1134	Chr16:89287514	1534	ChrX:102672840
335	Chr4:83772717	735	Chr10:9230683	1135	Chr17:136428	1535	ChrX:102882986
336	Chr4:84222554	736	Chr10:10085289	1136	Chr17:417927	1536	ChrX:102963690
337	Chr4:84853647	737	Chr10:11213124	1137	Chr17:902172	1537	ChrX:103031545
338	Chr4:86921324	738	Chr10:20232036	1138	Chr17:1050087	1538	ChrX:103041509
339	Chr4:91525055	739	Chr10:38431284	1139	Chr17:1080430	1539	ChrX:103045600
340	Chr4:140638437	740	Chr10:47149199	1140	Chr17:1092288	1540	ChrX:103222614
341	Chr4:146019630	741	Chr10:48888641	1141	Chr17:1106522	1541	ChrX:131895623
342	Chr4:152571678	742	Chr10:49430824	1142	Chr17:1204810	1542	ChrX:139399819
343	Chr4:157886451	743	Chr10:49917115	1143	Chr17:1278739	1543	ChrX:139513302
344	Chr4:164245781	744	Chr10:50543442	1144	Chr17:1279966	1544	ChrX:139600759
345	Chr4:170787793	745	Chr10:51008432	1145	Chr17:1281902	1545	ChrX:139732358
346	Chr4:176572999	746	Chr10:51565213	1146	Chr17:1411206	1546	ChrX:139766571
347	Chr4:177084250	747	Chr10:82031731	1147	Chr17:1556866	1547	ChrX:152111073
348	Chr4:177654730	748	Chr10:83108240	1148	Chr17:1608879	1548	ChrX:152430846
349	Chr4:178514650	749	Chr10:84718755	1149	Chr17:1655977	1549	ChrX:153059400
350	Chr4:179320089	750	Chr10:86184713	1150	Chr17:1677069	1550	ChrX:153137678
351	Chr4:180788739	751	Chr10:103197763	1151	Chr17:1713518	1551	ChrX:153183407
352	Chr4:184016512	752	Chr10:103251856	1152	Chr17:1780562	1552	ChrX:153289656
353	Chr4:184275328	753	Chr10:103312999	1153	Chr17:1824348	1553	ChrX:153303226
354	Chr4:184670317	754	Chr10:103355954	1154	Chr17:1887261	1554	ChrX:153325310
355	Chr4:185045337	755	Chr10:103408217	1155	Chr17:1985154	1555	ChrX:153357714
356	Chr4:186791884	756	Chr10:115430991	1156	Chr17:2194812	1556	ChrX:153523400
357	Chr4:188200282	757	Chr10:115821471	1157	Chr17:2586044	1557	ChrX:153577006

358	Chr4:188525699	758	Chr10:116361682	1158	Chr17:3303582	1558	ChrX:153629566
359	Chr4:190514286	759	Chr10:116464601	1159	Chr17:4483024	1559	ChrX:153671915
360	Chr4:190790761	760	Chr10:116584043	1160	Chr17:5542420	1560	ChrX:153737335
361	Chr5:465960	761	Chr10:117040968	1161	Chr17:7656113	1561	ChrX:153821275
362	Chr5:982577	762	Chr10:130134631	1162	Chr17:7724478	1562	ChrX:153940666
363	Chr5:1343480	763	Chr10:130743345	1163	Chr17:7793909	1563	ChrX:154213060
364	Chr5:1544526	764	Chr10:131190320	1164	Chr17:7889864	1564	ChrX:154275298
365	Chr5:1564717	765	Chr10:132159004	1165	Chr17:8128593	1565	ChrX:154305114
366	Chr5:1594141	766	Chr10:132339535	1166	Chr17:14249316	1566	ChrX:154445700
367	Chr5:1642656	767	Chr10:132829574	1167	Chr17:14667213	1567	ChrY:2654252
368	Chr5:1745156	768	Chr10:133989232	1168	Chr17:15133250	1568	ChrY:2655446
369	Chr5:2010192	769	Chr10:134305612	1169	Chr17:15154431	1569	ChrY:2734303
370	Chr5:2409887	770	Chr10:134424255	1170	Chr17:15168653	1570	ChrY:2802938
371	Chr5:2415030	771	Chr10:134490347	1171	Chr17:15405994	1571	ChrY:2838520
372	Chr5:3420912	772	Chr10:134576098	1172	Chr17:16878305	1572	ChrY:2873104
373	Chr5:3523855	773	Chr10:134765964	1173	Chr17:17398101	1573	ChrY:3447141
374	Chr5:5222923	774	Chr11:192261	1174	Chr17:17485643	1574	ChrY:5424369
375	Chr5:7709456	775	Chr11:203041	1175	Chr17:17991350	1575	ChrY:6899282
376	Chr5:9695958	776	Chr11:526562	1176	Chr17:18046136	1576	ChrY:7206281
377	Chr5:13391526	777	Chr11:682159	1177	Chr17:18111953	1577	ChrY:8590199
378	Chr5:15048168	778	Chr11:1319772	1178	Chr17:18156671	1578	ChrY:9645380
379	Chr5:18350670	779	Chr11:2439929	1179	Chr17:18193619	1579	ChrY:14607446
380	Chr5:36035656	780	Chr11:3629247	1180	Chr17:18761483	1580	ChrY:14789757
381	Chr5:36384448	781	Chr11:3825490	1181	Chr17:19619772	1581	ChrY:14821403
382	Chr5:36553056	782	Chr11:10209849	1182	Chr17:19700779	1582	ChrY:15016241
383	Chr5:36756645	783	Chr11:20210170	1183	Chr17:19727427	1583	ChrY:15831153
384	Chr5:38881715	784	Chr11:26682065	1184	Chr17:29159173	1584	ChrY:17028005
385	Chr5:45900759	785	Chr11:28303752	1185	Chr17:29422443	1585	ChrY:17530278
386	Chr5:50761205	786	Chr11:30033622	1186	Chr17:29576078	1586	ChrY:18930964
387	Chr5:88083621	787	Chr11:31429764	1187	Chr17:29664862	1587	ChrY:19357022
388	Chr5:88411097	788	Chr11:31632252	1188	Chr17:29865237	1588	ChrY:21153902
389	Chr5:88446397	789	Chr11:31663415	1189	Chr17:30179971	1589	ChrY:21868092
390	Chr5:88510176	790	Chr11:31711892	1190	Chr17:34956932	1590	ChrY:22922912
391	Chr5:88796625	791	Chr11:31746554	1191	Chr17:35442306	1591	ChrY:24354737
392	Chr5:89326286	792	Chr11:31814056	1192	Chr17:35722409	1592	ChrY:24875346
393	Chr5:89528228	793	Chr11:32138369	1193	Chr17:36046516	1593	ChrY:25276008

394	Chr5:89923291	794	Chr11:32303876	1194	Chr17:36076659	1594	ChrY:25341836
395	Chr5:90368358	795	Chr11:33913394	1195	Chr17:36099597	1595	ChrY:26191773
396	Chr5:90739192	796	Chr11:43150537	1196	Chr17:43785229	1596	ChrY:28789280
397	Chr5:107703571	797	Chr11:44284996	1197	Chr17:43865041	1597	1:145507646
398	Chr5:108133902	798	Chr11:44729728	1198	Chr17:43972615	1598	1:145507765
399	Chr5:108671068	799	Chr11:45245894	1199	Chr17:44017609	1599	4:1806119
400	Chr5:109202644	800	Chr11:45745904	1200	Chr17:44205592	1600	4:1807371
						1601	4:1803571
						1602	4:1807895
