# Supplementary material for: The implication of chromosomal abnormalities in the surgical outcomes of Chinese pediatric patients with congenital heart disease
Source: Front Cardiovasc Med. 2023 May 24;10:1164577. doi: 10.3389/fcvm.2023.1164577 (PMC10244782; doi:10.3389/fcvm.2023.1164577)
Supplement: Supplementary file 2 [file Datasheet2.pdf]

Supplementary Table S2. The HLPA assay involves 58 syndromes defined by DECIPHER database.

ID	Syndromes
1.1	1p36 microdeletion symdrome
1.2	Thrombocytopenia-Absent Radius（TAR）syndrome
1.3	1q21.1 recurrent microdeletion
1.3	1q21.1 recurrent microduplication
2.1	2p21 Microdeletion syndrome
2.2	2p15-16.1 microdeletion syndrome
2.3	2q33.1 deletion syndrome
2.4	2q37 monosomy
3.1	3q29 microdeletion syndrome，3q29 microduplication syndrome
4.1	Wolf-Hirschhorn Syndrome
5.1	Cri du Chat Syndrome
5.2	Familial Adenomatous Polyposis
5.3	Adult-onset autosomal dominant leukodystrophy（ADLD）
5.4	Sotos syndromes
7.1	Williams-Beuren Syndrome（WBS），17q11.23 duplication syndrome 22.2 22q11 deletion syndrome（Velocardiofacial/DiGeorge syndrome，22ql1 duplication syndrome
7.2	Split hand/foot malformation
8.1	8p23.1 deletion syndrome，8p23.1 duplication syndrome
8.2	8q21.11 Microdeletion Syndrome
9.1	9q subtelomeric deletion syndrome
11.1	WAGR 11p13 deletion syndrome
11.2	Potocki-Shaffer syndrome
12.1	12p13.33 Microdeletion Syndrome
12.2	12q14 microdeletion syndrome
15.1	Angelman syndrome（Type 1），Prader-Willi syndrome（Type 1）
15.2	Prader-Willi Syndrome（Type 2），Angelman syndrome（Type 2）
15.3	15q13.3 microdeletion syndrome
15.4	15q24 recurrent microdeletion syndrome
15.5	15q26 overgrowth syndrome
16.1	ATR-16 syndrome
16.2	Rubinstein-Taybi Syndrome
16.3	16p13.11 recurrent microdeletion，16p13.11 recurrent microduplication
16.4	16p11.2-p12.2 microduplication syndrome
16.5	16p11.2-p12.2 microdeletion syndrome
16.6	Recurrent 16p12.1 microdeletion
16.7	16p11.2 microduplication syndrome
17.1	Miller-Dieker syndrome (MDS)
17.2	Charcot-Marie-Tooth syndrome type 1A（CMT1A），Hereditary Liability to Pressure Palsies（HNPP）
17.3	Smith-Magenis Syndrome，Potocki-Lupski syndrome（17p11.2 duplication syndrome）
17.4	NF1-microdeleton syndrome
17.5	RCAD（renal cysts and diabetes）
17.6	l7q21.31 recurrent microdeletion syndrome（Koolen de Vries syndrome）
21.1	Early-onset Alzheimer disease with cerebral amyloid angiopathy
22.1	Cat-Eye Syndrome （Type I）
22.2	22q11 deletion syndrome (Velocardiofacila/DiGeorge syndrome), 22q11 duplication syndrome
22.3	22q11.2 distal deletion syndrome
22.4	22q13 deletion syndrome（Phelan-Mcdermid syndrome）
X.1	Leri-Weill dyschondrostosis（LWD）-SHOX deletion
X.2	Leri-Weill dyschondrostosis（LWD）-SHOX deletion
X.3	Steroid sulphatase deficiency（STS）
X.4	Xp11.22-p11.23 Microduplication
X.5	Xp11.22-linked intellectual disability
X.6	Pelizaeus-Merzbacher disease
X.7	Xq28（MECP2）duplication
X.8	Xq28 Microduplication
Y.1	AZFa deletion
Y.2	AZFa +AZFc deletion
Y.3	AZFb deletion
Y.4	AZFc deletion
