# Supplementary material for: The implication of chromosomal abnormalities in the surgical outcomes of Chinese pediatric patients with congenital heart disease
Source: Front Cardiovasc Med. 2023 May 24;10:1164577. doi: 10.3389/fcvm.2023.1164577 (PMC10244782; doi:10.3389/fcvm.2023.1164577)
Supplement: Supplementary file 3 [file Datasheet3.pdf]

Supplementary Table S3：Detailed information of the CNVs identinfied in 378 individuals.

ID	SEX	CNV	ANNOTATION	**ppCNV CNV carriers**
1	MALE	chr22: 19124024-21411092 deletion	22q11 microdeletion syndrome	yes
2	FEMALE	chr22: 20748457-21411092 deletion	22q11 microdeletion syndrome	yes
3	MALE	chrY: 24875782-26191389 deletion	benign	no
4	MALE	chr16: 218911-225106 duplication	HBA duplication，possible carriers or high-risk individuals for alphathalassaemia	no
5	FEMALE	Chr16:223651-225310 deletion，X:6456244-7867808 duplication	HBA deletion，possible carriers or high-risk individuals for alphathalassaemia	no
6	MALE	Chr6:931392-4426687 deletion，Chr16:223651-225310 deletion	likely pathogenic；HBA deletion，possible carriers or high-risk individuals for alpha	no
7	MALE	Chr22:19124024-21411092 deletion	22q11 microdeletion syndrome	yes
8	MALE	chrY: deletion（Y:24875782-Y:26191389）	benign	no
9	MALE	chrY:24875782-26191389 duplication	benign	no
10	MALE	Chr2:110873495-110954930 duplication	2q13(NPHP1)microduplication	no
11	MALE	Chr15:31324078-32440440 duplication	variants of uncertain significance	no
12	FEMALE	47,XXX	triple X syndrome	yes
13	MALE	Chr22:19124024-21411092 deletion，ChrY:25276257-26191389 deletion	22q11 microdeletion syndrome	yes
14	FEMALE	Chr2:110873495-110954930 deletion	benign	no
15	MALE	Chr1:145630220-145747034 deletion	1q21.1 susceptibility locus for Thrombocytopenia-Absent Radius (TAR) syndrome	yes
16	MALE	ChrY:25276257-26191389 deletion	benign	no
17	MALE	Chr16:218911-225310 deletion	HBA deletion，possible carriers or high-risk individuals for alphathalassaemia	no
18	MALE	Chr22:19124024-21411092 deletion	22q11 microdeletion syndrome	yes
19	MALE	Chr7:72858312-74071135 duplication；ChrY:25276257-26191389 deletion	7q11.23 duplication syndrome	yes
20	MALE	ChrY:25276257-26191389 deletion	benign	no
21	MALE	ChrY:25276257-26191389 duplication	benign	no
22	MALE	ChrY:25276257-26191389 deletion	benign	no
23	FEMALE	47,XXX	triple X syndrome	yes
24	FEMALE	Chr22:23022042-24106281 duplication	variants of uncertain significance	no
25	MALE	ChrY:25276257-26191389 deletion	benign	no
26	FEMALE	Chr18:72997477-77981673 deletion	likely pathogenic	yes
27	FEMALE	Chr22:19124024-21411092 deletion	22q11 microdeletion syndrome	yes
28	MALE	ChrY:24875782-26191389 deletion	benign	no
29	MALE	ChrY:24875782-26191389 duplication	benign	no
30	MALE	ChrY:25276257-26191389 deletion	benign	no
31	MALE	Chr16:15057818-16235196 duplication；ChrY:25276257-26191389 duplication	16p13.11 recurrent microduplication (neurocognitive disorder susceptibility locus);be	yes
32	MALE	ChrY:24875782-26191389 deletion	benign	no
33	MALE	Chr2:110873495-110954930 duplication，ChrY:24875782-26191389 deletion	2q13(NPHP1)microduplication；benign	no
34	MALE	Chr6:150863821-157410430 duplication，ChrY:24875782-26191389 deletion	likely pathogenic	yes
35	FEMALE	ChrX:591774-682004 deletion	Leri-Weill dyschondrostosis	yes
36	FEMALE	Chr2:110873495-110954930 duplication	2q13(NPHP1)microduplication	no
37	MALE	ChrY:25276257-26191389 duplication	benign	no
38	MALE	Chr22:19124024-21411092 deletion	22q11 microdeletion syndrome	yes
39	MALE	ChrY:24523602-24875782 deletion	benign	no
40	FEMALE	Chr2:110873495-110954930 deletion	benign	no
41	FEMALE	Chr2:110873495-110954930 duplication	2q13(NPHP1)microduplication	no
42	FEMALE	Chr16:218911-225310 deletion	HBA deletion，possible carriers or high-risk individuals for alphathalassaemia	no
43	FEMALE	Chr16:15057818-16235196 deletion	16p13.11 recurrent microdeletion (neurocognitive disorder susceptibility locus)	yes
44	MALE	Chr16:218911-225310 deletion	HBA deletion，possible carriers or high-risk individuals for alphathalassaemia	no
45	FEMALE	Chr22:19124024-21411092 deletion	22q11 microdeletion syndrome	yes
46	MALE	Chr22:19124024-21411092 deletion;chrY: duplication（Y:25276257-26191389）	22q11 microdeletion syndrome	yes
47	MALE	chrY: （Y:25276257-26191389) deletion	benign	no
48	MALE	chrY: （Y:24875782-26191389) deletion	benign	no
49	FEMALE	47,XX,+21	trisomy 21 syndrome	yes
50	FEMALE	Chr22：22343122-22536619 duplication	benign	no
51	FEMALE	Chr2:110873495-110954930 duplication	2q13(NPHP1)microduplication	no
52	FEMALE	Chr16:223651-225310 duplication	HBA duplication，possible carriers or high-risk individuals for alphathalassaemia	no
53	FEMALE	47,XX,+21	trisomy 21 syndrome	yes
54	FEMALE	Chr1:145630220-145747034 duplication	likely benign	no
55	MALE	ChrY:24875782-26191389 deletion	benign	no
56	FEMALE	Chr22:19124024-21411092 deletion	22q11 microdeletion syndrome	yes
57	MALE	ChrY:24875782-26191389 duplication	benign	no
58	FEMALE	Chr16:221000-223651 duplication	HBA duplication，possible carriers or high-risk individuals for alphathalassaemia	no
59	MALE	Chr22:19124024-21411092 duplication	22q11 microduplication syndrome	yes
60	MALE	ChrY:25276257-26191389 duplication	benign	no
61	FEMALE	Chr2:110873495-110954930 duplication	2q13(NPHP1) microduplication	no
62	MALE	ChrY:24875782-26191389 deletion	benign	no
63	FEMALE	Chr16: 223651-225310 deletion	HBA deletion，possible carriers or high-risk individuals for alphathalassaemia	no
64	MALE	Chr7:72858312-74071135 deletion	Williams-Beuren syndrome	yes
65	MALE	Chr2:110873495-110954930 duplication	2q13(NPHP1)microduplication	no
66	MALE	Chr2:110873495-110954930 duplication	2q13(NPHP1)microduplication	no
67	FEMALE	47，XX,+21	trisomy 21 syndrome	yes
68	MALE	Chr16:28846059-29008389 duplication	variants of uncertain significance	no
69	FEMALE	Chr15:31324078-31570595 duplication	variants of uncertain significance	no
70	FEMALE	47,XY,+21	trisomy 21 syndrome	yes
71	FEMALE	Chr7:72858312-74071135 deletion	Williams-Beuren syndrome	yes
72	MALE	Chr22:22343122-22536619 duplication	benign	no
73	FEMALE	Chr22:19124024-21411092 deletion	22q11 microdeletion syndrome	yes
74	MALE	Chr15:22820345-23300217 deletion	15q11.2 microdeletion syndrome	yes
75	MALE	ChrX:591774-2138466 duplication，ChrY:2654617-28789286 duplication	XYY syndrome	yes
76	MALE	Chr1:243782460-245028377 deletion，ChrY:25276257-26191389 deletion	likely pathogenic；benign	yes
77	MALE	Chr22:19124024-21411092 deletion	22q11 microdeletion syndrome	yes
78	MALE	Chr22:19124024-20094119 deletion，ChrY:25276257-26191389 duplication	22q11 microdeletion syndrome；benign	yes
79	FEMALE	Chr22:19124024-21411092 deletion	22q11 microdeletion syndrome	yes
80	MALE	Chr22:22343122-22536619 duplication	benign	no
81	MALE	Chr2:110873495-110954930 duplication	2q13(NPHP1)microduplication	no
82	MALE	Chr22:19124024-21411092 deletion，ChrY:25276257-26191389 deletion	22q11 microdeletion syndrome；benign	yes
83	MALE	Chr7:5732486-5835557 duplication	variants of uncertain significance	no
84	MALE	ChrY:25276257-26191389 deletion	benign	no
85	MALE	ChrY:24875782-26191389 duplication	benign	no
86	MALE	ChrY:25276257-26191389 deletion	benign	no
87	MALE	Chr22:19124024-21411092 deletion	22q11 microdeletion syndrome	yes
88	MALE	Chr22:19124024-21411092 deletion	22q11 microdeletion syndrome	yes
89	FEMALE	Chr15:32149039-32440440 duplication	benign	no
90	MALE	ChrY:25276257-26191389 deletion	benign	no
91	MALE	ChrY:25276257-26191389 deletion	benign	no
92	MALE	ChrY:25276257-26191389 deletion	benign	no
93	FEMALE	Chr15:22820345-23024174 duplication	benign	no
94	MALE	ChrY:24875782-26191389 deletion	benign	no
95	MALE	Chr1:146627509-147363445 duplication;ChrY:25276257-26191389 deletion	1q21.1 microduplication；benign	yes
96	MALE	Chr22:20748457-21411092 deletion	22q11 microdeletion syndrome	yes
97	FEMALE	Chr16:221000-223651 duplication	HBA duplication，possible carriers or high-risk individuals for alphathalassaemia	no
98	MALE	ChrY:24523602-25341856 deletion	benign	no
99	MALE	Duplication of sex chromosome homologous regions：ChrX:591774-846100 or ChrY：541	benign	no
100	FEMALE	Chr2:339675-855239 duplication	benign	no
101	MALE	Chr16:223651-225310 deletion	HBA deletion，possible carriers or high-risk individuals for alphathalassaemia	no
102	MALE	ChrY:25276257-26191389 duplication	benign	no
103	MALE	ChrY:25276257-26191389 deletion	benign	no
104	MALE	47,XY,+21	trisomy 21 syndrome	yes
105	MALE	Chr17:14249295-15405970 deletion	Hereditary Liability to Pressure Palsies（HNPP）	yes
106	FEMALE	45,XO	XO syndrome	yes
107	FEMALE	ChrX:6456244-7867808 duplication	variants of uncertain significance	no

108	MALE	Chr16:223651-225310 deletion;ChrY:25276257-26191389 deletion	HBA deletion，possible carriers or high-risk individuals for alphathalassaemia;benig	no
109	FEMALE	47,XX,+21; Chr2:110873495-110954930 duplication	trisomy 21 syndrome；2q13(NPHP1)microduplication	yes
110	FEMALE	Chr1:145630220-145747034 duplication	likely benign	no
111	MALE	Chr22:22343122-22536619 duplication	benign	no
112	FEMALE	Chr16:221000-223651 duplication	HBA duplication，possible carriers or high-risk individuals for alphathalassaemia	no
113	MALE	Chr16:223651-225310 duplication;ChrY:25276257-26191389 deletion	HBA duplication，possible carriers or high-risk individuals for alphathalassaemia;be	no
114	MALE	Chr22:19124024-21411092 duplication	22q11 microduplication syndrome	yes
115	MALE	Chr7:72858312-74071135 deletion	Williams-Beuren syndrome	yes
116	MALE	Chr1:146627509-147363445 duplication	1q21.1 microduplication syndrome	yes
117	MALE	ChrY:25276257-26191389 deletion	benign	no
118	FEMALE	47,XXX	triple X syndrome	yes
119	MALE	47,XY,+21	trisomy 21 syndrome	yes
120	FEMALE	Chr2:60386223-61708372 duplication	likely pathogenic	yes
121	MALE	ChrY:25276257-26191389 deletion	benign	no
122	FEMALE	Chr2:110873495-110954930 duplication	2q13(NPHP1)microduplication	no
123	MALE	Chr2:110873495-110954930 duplication	2q13(NPHP1)microduplication	no
124	MALE	Chr16:21995515-22256000 duplication	variants of uncertain significance	no
125	FEMALE	Chr4:101768-13542552 deletion	likely pathogenic	yes
126	FEMALE	Chr15:32066389-32440440 duplication	benign	no
127	MALE	Chr2:110873495-110954930 duplication;ChrY:25276257-26191389 deletion	2q13(NPHP1)microduplication ;benign	no
128	FEMALE	Chr15:22820345-23024174 deletion	benign	no
129	MALE	Chr22:16868894-17305107 duplication	variants of uncertain significance	no
130	FEMALE	Chr2:110873495-110954930 duplication	2q13(NPHP1)microduplication	no
131	MALE	47,XY,+21	trisomy 21 syndrome	yes
132	FEMALE	Chr16:223651-225310 duplication	HBA duplication，possible carriers or high-risk individuals for alphathalassaemia	no
133	FEMALE	Chr7:72858312-74071135 deletion	Williams-Beuren syndrome	yes
134	MALE	Chr16:223651-225310 deletion	HBA deletion，possible carriers or high-risk individuals for alphathalassaemia	no
135	MALE	Chr16:221000-223651 deletion	HBA deletion，possible carriers or high-risk individuals for alphathalassaemia	no
136	MALE	ChrY:24875782-26191389 duplication	benign	no
137	MALE	Chr2:110873495-110954930 duplication;ChrX:6456244-7151842 duplication;ChrY:2487	2q13(NPHP1)microduplication ;benign；benign	no
138	FEMALE	Chr1:864330-1860755 deletion;Chr17:80390309-81152030 duplication	likely pathogenic；variants of uncertain significance	yes
139	FEMALE	Chr15:22820345-23300217 duplication	benign	no
140	MALE	Chr11:116871491-134444167 duplication;Chr22:16868894-20094119 duplication	likely pathogenic；likely pathogenic	yes
141	FEMALE	Chr15:32066389-32440440 duplication	benign	no
142	MALE	Chr16:221000-223651 duplication	HBA duplication，possible carriers or high-risk individuals for alphathalassaemia	no
143	MALE	47,XXY	XXY syndrome	yes
144	MALE	Chr16:223651-225310 deletion	HBA deletion，possible carriers or high-risk individuals for alphathalassaemia	no
145	FEMALE	Chr2:110873495-110954930 duplication	2q13(NPHP1)microduplication	no
146	FEMALE	Chr1:146627509-147363445 duplication	1q21.1 microduplication syndrome	yes
147	FEMALE	47,XX,+21	trisomy 21 syndrome	yes
148	FEMALE	Chr16:223651-225310 duplication	HBA duplication，possible carriers or high-risk individuals for alphathalassaemia	no
149	FEMALE	Chr16:218911-227498 deletion	HBA deletion，possible carriers or high-risk individuals for alphathalassaemia	no
150	MALE	Chr2:110873495-110954930 duplication	2q13(NPHP1)microduplication	no
151	FEMALE	Chr16:221000-223651 duplication	HBA duplication，possible carriers or high-risk individuals for alphathalassaemia	no
152	MALE	ChrY:24875782-26191389 deletion	benign	no
153	MALE	ChrY:25276257-26191389 duplication	benign	no
154	MALE	Chr2:110873495-110954930 duplication,ChrY:25276257-26191389 duplication	2q13(NPHP1)microduplication ;benign	no
155	FEMALE	45,XO	XO syndrome	yes
156	FEMALE	Chr10:103197740-117040946 duplication	likely pathogenic	yes
157	MALE	Chr11:116871491-134604970 duplication;Chr22:16868894-20094119 duplication	likely pathogenic；likely pathogenic	yes
158	MALE	ChrY:25276257-26191389 deletion	benign	no
159	FEMALE	47,XX,+21	trisomy 21 syndrome	yes
160	MALE	Duplication of sex chromosome homologous regions： ChrX:591774-682004 or ChrY:541	benign	no
161	MALE	Duplication of sex chromosome homologous regions：ChrX:811390-846100 or ChrY:7613	benign	no
162	FEMALE	Chr15:22820345-23300217 duplication	benign	no
163	FEMALE	Chr20:62080193-62643052 deletion	variants of uncertain significance	no
164	FEMALE	Duplication of sex chromosome homologous regions：ChrX:811390-846100	benign	no
165	FEMALE	Chr16:223651-225310 deletion	HBA deletion，possible carriers or high-risk individuals for alphathalassaemia	no
166	FEMALE	47,XX,+21;Chr2:110873495-110954930 duplication	trisomy 21 syndrome；2q13(NPHP1)microduplication	yes
167	MALE	47,XXY; Chr16:223651-225310 duplication	XXY syndrome；HBA duplication，possible carriers or high-risk individuals for alp	yes
168	MALE	Chr16:221000-223651 duplication	HBA duplication，possible carriers or high-risk individuals for alphathalassaemia	no
169	FEMALE	Chr15:22820345-23024174 deletion	benign	no
170	MALE	Chr15:22820345-23300217 deletion	15q11.2 microdeletion syndrome	yes
171	MALE	Chr7:72858312-74071135 deletion	Williams-Beuren syndrome	yes
172	FEMALE	Chr2:96754320-97529420 deletion	variants of uncertain significance	no
173	FEMALE	Chr2:110873495-110932071 duplication	benign	no
174	MALE	Chr16:218911-227498 deletion	HBA deletion，possible carriers or high-risk individuals for alphathalassaemia	no
175	FEMALE	Chr7:72858312-74071135 deletion	Williams-Beuren syndrome	yes
176	MALE	Chr22:17447338-18031485 duplication	variants of uncertain significance	no
177	FEMALE	Chr22:16868894-17466043 duplication	benign	no
178	FEMALE	Chr2:110873495-110954930 duplication	2q13(NPHP1)microduplication	no
179	MALE	47,XY,+21	trisomy 21 syndrome	yes
180	FEMALE	Chr16:223651-225310 deletion	HBA deletion，possible carriers or high-risk individuals for alphathalassaemia	no
181	FEMALE	Chr22:16868894-17466043 duplication	benign	no
182	MALE	Chr22:19124024-21411092 deletion	22q11 microdeletion syndrome	yes
183	MALE	Chr15:32066389-32440440 duplication	benign	no
184	MALE	Chr16:221000-223651 duplication	HBA duplication，possible carriers or high-risk individuals for alphathalassaemia	no
185	MALE	ChrX:77193983-77284129 duplication	variants of uncertain significance	no
186	MALE	Chr16:21995515-22826377 duplication	variants of uncertain significance	no
187	FEMALE	Chr22:22343122-22536619 duplication	benign	no
188	MALE	47,XX,+21	trisomy 21 syndrome	yes
189	FEMALE	Chr2:110873495-110954930 duplication	2q13(NPHP1)microduplication	no
190	MALE	Chr16:15057818-16235196 duplication	16p13.11 recurrent microduplication (neurocognitive disorder susceptibility locus)	yes
191	MALE	Chr16:223651-225310 deletion	HBA deletion，possible carriers or high-risk individuals for alphathalassaemia	no
192	FEMALE	Chr2:110873495-110954930 duplication	2q13(NPHP1)microduplication	no
193	MALE	Chr16:221000-223651 duplication	HBA duplication，possible carriers or high-risk individuals for alphathalassaemia	no
194	MALE	Chr15:32066389-32440440 duplication	benign	no
195	FEMALE	Chr15:22820345-23300217 duplication;Chr22:19124024-21411092 deletion	benign；22q11 microdeletion syndrome	yes
196	MALE	Chr16:221000-223651 deletion	HBA deletion，possible carriers or high-risk individuals for alphathalassaemia	no
197	FEMALE	Chr16:223651-225310 deletion	HBA deletion，possible carriers or high-risk individuals for alphathalassaemia	no
198	FEMALE	Chr22:22343122-22536619 duplication	benign	no
199	FEMALE	Chr4:1875104-1957875 deletion	variants of uncertain significance	no
200	MALE	Chr2:110873495-110954930 duplication	2q13(NPHP1)microduplication	no
201	MALE	Chr22:19124024-21411092 deletion	22q11 microdeletion syndrome	yes
202	MALE	Chr16:218911-227498 deletion	HBA deletion，possible carriers or high-risk individuals for alphathalassaemia	no
203	FEMALE	Chr16:15057818-16235196 deletion	likely pathogenic	yes
204	MALE	47,XY,+21	trisomy 21 syndrome	yes
205	MALE	Chr16:223651-225310 duplication	HBA duplication，possible carriers or high-risk individuals for alphathalassaemia	no
206	FEMALE	Chr16:221000-223651 deletion	HBA deletion，possible carriers or high-risk individuals for alphathalassaemia	no
207	FEMALE	47,XX,+21	trisomy 21 syndrome	yes
208	FEMALE	ChrX:6456244-7867808 deletion	likely pathogenic	yes
209	FEMALE	Chr16:221000-223651 duplication	HBA duplication，possible carriers or high-risk individuals for alphathalassaemia	no
210	MALE	Chr22:22343122-22536619 duplication	benign	no
211	MALE	47,XY,+21	trisomy 21 syndrome	yes
212	MALE	Chr2:110873495-110954930 deletion	benign	no
213	FEMALE	Chr22:22343122-22536619 duplication	benign	no
214	MALE	Chr16:221000-223651 duplication	HBA duplication，possible carriers or high-risk individuals for alphathalassaemia	no
215	FEMALE	Chr16:221000-223651 duplication;Chr22:19124024-21366404 duplication	HBA duplication，possible carriers or high-risk individuals for alphathalassaemia;22	yes
216	MALE	Chr22:22343122-22536619 duplication	benign	no

217	MALE	Chr2:110873495-110954930 deletion	benign	no
218	MALE	Chr2:110873495-110954930 duplication;Chr16:223651-225310 duplication	2q13(NPHP1)microduplication；HBA duplication，possible carriers or high-risk in	no
219	MALE	Chr16:218911-227498 deletion	HBA deletion，possible carriers or high-risk individuals for alphathalassaemia	no
220	MALE	47,XY,+21	trisomy 21 syndrome	yes
221	MALE	Chr16:223651-225310 duplication	HBA duplication，possible carriers or high-risk individuals for alphathalassaemia	no
222	FEMALE	Chr11:130848693-134604970 deletion	likely pathogenic	yes
223	FEMALE	Chr17:34956912-36099575 deletion	Miller-Dieker syndrome	yes
224	MALE	Chr7:72858312-74071135 deletion	Williams-Beuren syndrome	yes
225	FEMALE	Chr15:31324078-32440440 deletion	15q13.3 microdeletion syndrome	yes
226	FEMALE	Chr22:19745034-21098944 deletion	22q11 microdeletion syndrome	yes
227	FEMALE	47,XXX	triple X syndrome	yes
228	FEMALE	Chr22:19124024-21411092 deletion	22q11 microdeletion syndrome	yes
229	FEMALE	Chr2:110873495-110954930 duplication;Chr16:223651-225310 duplication	2q13(NPHP1)microduplication；HBA duplication，possible carriers or high-risk in	no
230	MALE	Chr7:72858312-74071135 deletion	Williams-Beuren syndrome	yes
231	MALE	Chr15:31460682-36872001 duplication	likely pathogenic	yes
232	MALE	Chr22:19124024-21411092 deletion	22q11 microdeletion syndrome	yes
233	FEMALE	Chr22:19124024-21411092 deletion	22q11 microdeletion syndrome	yes
234	MALE	Chr7:72858312-74071135 deletion	Williams-Beuren syndrome	yes
235	MALE	Chr17:8128572-15405970 duplication	likely pathogenic	yes
236	FEMALE	Chr17:1713495-1824327 duplication	variants of uncertain significance	no
237	FEMALE	Chr16:218911-227498 deletion	HBA deletion，possible carriers or high-risk individuals for alphathalassaemia	no
238	FEMALE	Chr15:22820345-23300217 duplication	benign	no
239	MALE	Chr7:158734763-159029030 duplication;Chr16:221000-223651 deletion	variants of uncertain significance；HBA deletion，possible carriers or high-risk indi	no
240	FEMALE	ChrX:591774-48316953 deletion	likely pathogenic	yes
241	FEMALE	Chr16:223651-225310 deletion	HBA deletion，possible carriers or high-risk individuals for alphathalassaemia	no
242	MALE	Chr16:221000-223651 deletion	HBA deletion，possible carriers or high-risk individuals for alphathalassaemia	no
243	MALE	Chr16:221000-223651 duplication	HBA duplication，possible carriers or high-risk individuals for alphathalassaemia	no
244	FEMALE	Chr16:223651-225310 deletion	HBA deletion，possible carriers or high-risk individuals for alphathalassaemia	no
245	MALE	Chr16:223651-225310 deletion	HBA deletion，possible carriers or high-risk individuals for alphathalassaemia	no
246	FEMALE	Chr7:72858312-74071135 deletion	Williams-Beuren syndrome	yes
247	FEMALE	47,XX,+21; Chr15:22820345-23300217 duplication	trisomy 21 syndrome；benign	yes
248	FEMALE	Chr16:28846059-29008389 deletion	likely pathogenic	yes
249	MALE	Chr2:110873495-110954930 duplication	2q13(NPHP1)microduplication	no
250	MALE	Chr1:238535910-238816971 duplication;Chr16:223651-225310 duplication	likely pathogenic;HBA duplication，possible carriers or high-risk individuals for alp	yes
251	MALE	Chr16:223651-225310 deletion	HBA deletion，possible carriers or high-risk individuals for alphathalassaemia	no
252	MALE	Chr2:110873495-110954930 duplication	2q13(NPHP1)microduplication	no
253	MALE	Chr22:19124024-21411092 deletion	22q11 microdeletion syndrome	yes
254	FEMALE	47,XX,+21	trisomy 21 syndrome	yes
255	FEMALE	Chr22:19124024-21411092 deletion	22q11 microdeletion syndrome	yes
256	FEMALE	Chr15:32066389-32410665 duplication;Chr16:223651-225310 duplication	benign；HBA duplication，possible carriers or high-risk individuals for alphathalas	no
257	MALE	Chr16:28846059-29008389 deletion	likely pathogenic	yes
258	MALE	Chr1:243782460-246739575 deletion	pathogenic	yes
259	FEMALE	Chr22:22343122-22536619 duplication	benign	no
260	MALE	Chr15:32066389-32440440 duplication	benign	no
261	MALE	Chr2:44413021-44575611 deletion	2p21 microdeletion syndrome	yes
262	FEMALE	47,XX,+21; Chr22:22343122-22536619 duplication	trisomy 21 syndrome；benign	yes
263	FEMALE	45,XO	XO syndrome	yes
264	FEMALE	Chr2:110873495-110954930 duplication	2q13(NPHP1)microduplication	no
265	FEMALE	Chr22:19124024-21411092 duplication	22q11microduplication syndrome	yes
266	MALE	Chr16:223651-225310 deletion	HBA deletion，possible carriers or high-risk individuals for alphathalassaemia	no
267	FEMALE	45,XO	XO syndrome	yes
268	MALE	Chr16:221000-223651 deletion	HBA deletion，possible carriers or high-risk individuals for alphathalassaemia	no
269	FEMALE	Chr1:145630220-145747034 deletion	1q21.1 susceptibility locus for Thrombocytopenia-Absent Radius (TAR) syndrome	yes
270	FEMALE	Chr16:218911-223651 duplication	HBA duplication，possible carriers or high-risk individuals for alphathalassaemia	no
271	FEMALE	47,XX,+21	trisomy 21 syndrome	yes
272	FEMALE	Chr16:218911-223651 duplication	HBA duplication，possible carriers or high-risk individuals for alphathalassaemia	no
273	MALE	Chr22:19124024-21411092 deletion	22q11 microdeletion syndrome	yes
274	FEMALE	Chr15:32066389-32440440 duplication	benign	no
275	MALE	Chr2:110873495-110954930 duplication；Chr15:74627382-75706577 deletion	2q13(NPHP1)microduplication；15q24 microdeletion syndrome	yes
276	FEMALE	Chr16:218911-227498 deletion	HBA deletion，possible carriers or high-risk individuals for alphathalassaemia	no
277	MALE	Chr7:139121221-159029030 duplication;Chr14:21574805-21996913 duplication	pathogenic；variants of uncertain significance	yes
278	FEMALE	Chr1:145438511-145747034 duplication	benign	no
279	MALE	ChrX:6456244-7867808 duplication	variants of uncertain significance	no
280	FEMALE	Chr22:21098944-21411092 deletion	variants of uncertain significance	no
281	MALE	Chr7:72858312-74071135 deletion	Williams-Beuren syndrome	yes
282	FEMALE	Chr2:110873495-110954930 duplication	2q13(NPHP1)microduplication	no
283	FEMALE	ChrX:6456244-7867808 duplication	variants of uncertain significance	no
284	MALE	47, +21	trisomy 21 syndrome	yes
285	FEMALE	Chr16:223651-225310 deletion	HBA deletion，possible carriers or high-risk individuals for alphathalassaemia	no
286	MALE	Chr16:28846059-29008389 deletion	likely pathogenic	yes
287	FEMALE	Chr1:146627509-147363445 duplication	1q21.1 microduplication	yes
288	MALE	Chr8:11617710-11696284 duplication	variants of uncertain significance	no
289	MALE	Chr22:22343122-22536619 duplication	benign	no
290	MALE	Duplication of sex chromosome homologous regions：ChrX:591774-846100 or ChrY:5417	benign	no
291	FEMALE	Chr2:110873495-110954930 deletion	benign	no
292	MALE	Chr2:110873495-110954930 duplication	2q13(NPHP1)microduplication	no
293	MALE	Chr8:8244906-11696284 deletion	8p23.1 microdeletion syndrome	yes
294	MALE	Chr22:19124024-21411092 deletion	22q11 microdeletion syndrome	yes
295	MALE	Chr2:110873495-110954930 deletion	benign	no
296	FEMALE	Chr16:223651-225310 deletion	HBA deletion，possible carriers or high-risk individuals for alphathalassaemia	no
297	FEMALE	Chr22:20748457-21411092 deletion	22q11 microdeletion syndrome	yes
298	FEMALE	Chr16:221000-223651 deletion	HBA deletion，possible carriers or high-risk individuals for alphathalassaemia	no
299	MALE	Chr7:72858312-74071135 deletion	Williams-Beuren syndrome	yes
300	FEMALE	Chr15:22820345-23300217 deletion	15q11.2 microdeletion syndrome	yes
301	MALE	Chr8:413101-6302778 deletion	pathogenic	yes
302	FEMALE	Chr15:22820345-23300217 deletion	15q11.2 microdeletion syndrome	yes
303	FEMALE	ChrX:591774-682004 duplication	benign	no
304	MALE	Chr22:19124024-21411092 deletion	22q11 microdeletion syndrome	yes
305	MALE	Chr10:134424234-134765940 duplication;Chr16:223651-225310 deletion	benign；HBA duplication，possible carriers or high-risk individuals for alphathalas	no
306	FEMALE	Chr1:145438511-145747034 deletion;Chr22:19124024-21411092 deletion	22q11 microdeletion syndrome	yes
307	MALE	Chr16:221000-223651 duplication	HBA duplication，possible carriers or high-risk individuals for alphathalassaemia	no
308	FEMALE	ChrX:6456244-7867808 duplication	variants of uncertain significance	no
309	FEMALE	Chr2:110873495-110954930 duplication	2q13(NPHP1)microduplication	no
310	MALE	Chr14:105643861-106153701 duplication	variants of uncertain significance	no
311	FEMALE	Chr22:19124024-21411092 duplication	22q11microduplication syndrome	yes
312	FEMALE	47,XX,+21	trisomy 21 syndrome	yes
313	FEMALE	Chr2:110873495-110954930 duplication	2q13(NPHP1)microduplication	no
314	FEMALE	Chr2:110873495-110954930 duplication	2q13(NPHP1)microduplication	no
315	FEMALE	Chr16:15057818-15507525 duplication	benign	no
316	FEMALE	Chr5:465393-18350650 deletion	Cri du Chat syndrome	yes
317	MALE	Chr15:32066389-32440440 duplication	benign	no
318	FEMALE	Chr2:110873495-110954930 duplication	2q13(NPHP1)microduplication	no
319	MALE	Chr16:221000-223651 deletion	HBA deletion，possible carriers or high-risk individuals for alphathalassaemia	no
320	FEMALE	Chr1:145630220-145747034 deletion	1q21.1 susceptibility locus for Thrombocytopenia-Absent Radius (TAR) syndrome	yes
321	FEMALE	47,XX,+21	trisomy 21 syndrome	yes
322	FEMALE	Chr7:72858312-74071135 deletion	Williams-Beuren syndrome	yes
323	MALE	Chr16:28846059-29008389 deletion	likely pathogenic	yes
324	FEMALE	Chr16:28846059-29008389 deletion	likely pathogenic	yes
325	MALE	Chr16:15166722-16235196 duplication	16p13.11 recurrent microduplication (neurocognitive disorder susceptibility locus)	yes

326	FEMALE	Chr16:21995515-22256000 duplication	variants of uncertain significance	no
327	FEMALE	Chr2:110873495-110954930 duplication	2q13(NPHP1)microduplication	no
328	FEMALE	Chr15:32376176-32440440 duplication	benign	no
329	FEMALE	47,XX,+21	trisomy 21 syndrome	yes
330	FEMALE	Chr2:186896862-187031445 deletion	variants of uncertain significance	no
331	FEMALE	Chr7:5732486-5835557 duplication	variants of uncertain significance	no
332	FEMALE	Chr16:223651-225310 deletion	HBA deletion，possible carriers or high-risk individuals for alphathalassaemia	no
333	MALE	Chr7:72858312-74071135 deletion;Duplication of sex chromosome homologous regions：	Williams-Beuren syndrome	yes
334	FEMALE	Chr2:110873495-110954930 duplication	2q13(NPHP1)microduplication	no
335	MALE	Chr2:110873495-110954930 deletion	benign	no
336	FEMALE	Chr22:19124024-21411092 deletion	22q11 microdeletion syndrome	yes
337	FEMALE	Chr1:145438511-145747034 deletion	1q21.1 susceptibility locus for Thrombocytopenia-Absent Radius (TAR) syndrome	yes
338	MALE	Chr2:110873495-110954930 duplication	2q13(NPHP1)microduplication	no
339	MALE	Chr2:110873495-110954930 deletion	benign	no
340	FEMALE	Chr22:22343122-22536619 duplication	benign	no
341	MALE	Chr15:22820345-23024174 duplication	benign	no
342	MALE	Chr16:218911-227498 deletion	HBA deletion，possible carriers or high-risk individuals for alphathalassaemia	no
343	FEMALE	Chr8:413101-6302778 deletion;Chr15:72923127-101887270 duplication	pathogenic；pathogenic	yes
344	MALE	Chr16:223618-225336 deletion	HBA deletion，possible carriers or high-risk individuals for alphathalassaemia	no
345	FEMALE	Chr1:145630244-145747058 deletion	variants of uncertain significance	no
346	MALE	Chr2:96754344-97529813和Chr16:223618-225336 deletion	variants of uncertain significance；HBA deletion，possible carriers or high-risk indi	no
347	MALE	Chr1:146627532-147293834 duplication	variants of uncertain significance	no
348	MALE	Chr22:19124045-21411131 deletion	22q11 microdeletion syndrome	yes
349	FEMALE	Chr17:14249316-15405994 duplication	Charcot-Marie-Tooth syndrome	yes
350	FEMALE	Chr17:14249316-15405994 duplication	Charcot-Marie-Tooth syndrome	yes
351	MALE	Chr7:72858332-74071238 deletion	Williams-Beuren syndrome	yes
352	FEMALE	Chr22:19124045-21411131 deletion	22q11 microdeletion syndrome	yes
353	FEMALE	Chr22:20748477-23635413 deletion	22q11.2 deletion syndrome	yes
354	MALE	Chr22:20094141-21976318 duplication	22q11duolication syndrome	yes
355	MALE	Chr5:175813609-176165224 duplication;Chr22:19124045-21411131 deletion	22q11 microdeletion syndrome	yes
356	MALE	Chr21:15480245-48089327 duplication	pathogenic	yes
357	FEMALE	Chr9:130742496-141008509 duplication	pathogenic	yes
358	MALE	Chr16:223618-225336 deletion	HBA deletion，possible carriers or high-risk individuals for alphathalassaemia	no
359	FEMALE	Chr16:15623658-16235219 deletion	likely pathogenic	yes
360	MALE	Chr16:29674343-30157283 deletion	likely pathogenic	yes
361	FEMALE	Chr7:72858332-74071238 deletion	Williams-Beuren syndrome	yes
362	FEMALE	Chr21:15480245-48089327 duplication	pathogenic	yes
363	MALE	ChrY:25276257-26191389 duplication	benign	no
364	MALE	Chr7:72858312-74071135 deletion	Williams-Beuren syndrome	yes
365	MALE	Chr7:72858312-74071135 deletion	Williams-Beuren syndrome	yes
366	FEMALE	Chr17:136407-1281875 deletion	Miller-Dieker syndrome	yes
367	MALE	Chr2:110873495-110954930 duplication，ChrY:25276257-26191389 duplication	2q13(NPHP1)microduplication；benign	no
368	FEMALE	Chr16:218911-227498 deletion	HBA deletion，possible carriers or high-risk individuals for alphathalassaemia	no
369	FEMALE	Chr3:195923166-197299885 deletion	3q29 microdeletion syndrome	yes
370	MALE	Chr7:72858312-74071135 deletion	Williams-Beuren syndrome	yes
371	MALE	Chr7:72858312-74071135 deletion	Williams-Beuren syndrome	yes
372	MALE	Chr16:21995536-22256022 deletion	16p12.1 microdeletion syndrome	yes
373	FEMALE	Chr9:470059-708625 duplication	variants of uncertain significance	no
374	MALE	Chr1:864351-4529446 deletion	pathogenic	yes
375	FEMALE	Chr22:19124045-21411131 deletion	22q11 microdeletion syndrome	yes
376	MALE	Chr16:223618-225336 deletion	HBA deletion，possible carriers or high-risk individuals for alphathalassaemia	no
377	FEMALE	Chr22:21098965-21411131 deletion	variants of uncertain significance	no
378	MALE	Chr7:72858332-74071238 deletion	Williams-Beuren syndrome	yes
