# Supplementary material for: The implication of chromosomal abnormalities in the surgical outcomes of Chinese pediatric patients with congenital heart disease
Source: Front Cardiovasc Med. 2023 May 24;10:1164577. doi: 10.3389/fcvm.2023.1164577 (PMC10244782; doi:10.3389/fcvm.2023.1164577)
Supplement: Supplementary file 4 [file Datasheet4.pdf]

Supplementary Table S4.Frequency of ppCNVs in Han Chinese population of DGV database.

Chr	start	end	type	Number of carriers in Han Chinese of DGV	Carriers in Han Chinese of DGV	Number of non-carriers in Han Chinese of DGV	frequency
15	22820345	23300217	Loss	1	NA18555	302	0.0033003
2	110873495	110954930	Gain	5	NA18629,HG00581,NA18558,NA18553,NA1862	298	0.0165017
16	221000	223651	Gain	5	HG00699,HG00728,HG00684,HG00729,HG0069	298	0.0165017
16	223651	225310	Gain	5	HG00699,HG00728,HG00684,HG00729,HG0069	298	0.0165017
