# Supplementary material for: The implication of chromosomal abnormalities in the surgical outcomes of Chinese pediatric patients with congenital heart disease
Source: Front Cardiovasc Med. 2023 May 24;10:1164577. doi: 10.3389/fcvm.2023.1164577 (PMC10244782; doi:10.3389/fcvm.2023.1164577)
Supplement: Supplementary file 5 [file Datasheet5.pdf]

Supplementary Table S5. Baseline characteristics of CHD patients (stratified by the presence or absence of CNV)

Characteristics		CNV preset	CNV absent	Combined	P-value
Sample size		378	1384	1762	
Male, %		204 (53.97%)	694 (50.14%)	898 (51.96%)	0.188
Female, %		174 (46.03%)	690 (49.86%)	864 (48.04%)	
Age, months		24.33 ± 30.28	25.12 ± 30.92	24.95 ± 30.78	0.420
Weight, kg		11.00 ± 7.43	11.50 ± 7.64	11.393 ± 7.60	0.254
Complex surgery, %					
	YES	184 (48.68%)	519 (37.50%)	703 (39.90%)	**<0.001**
	NO	193 (51.32%)	865 (62.50%)	1058 (60.10%)	
CPBT, min		70.23 ± 50.43	62.28 ± 41.72	63.99 ± 43.84	**0.005**
ACCT, min		37.70 ± 29.95	32.97 ± 25.51	33.99± 26.58	**0.005**
MVT, min		74.57 ± 207.60	51.43 ± 134.96	56.38 ± 153.66	**0.009**
Delayed sternal closure, %					
	YES	10 (2.6%)	37 (2.67%)	47 (2.67%)	0.976
	NO	368(97.4%)	1347 (97.33%)	1715 (97.33%)	
Hemostasis, %					
	YES	4 (1.06%)	5 (0.36%)	9 (0.51%)	0.092
	NO	374 (98.94%)	1379 (99.64%)	1753 (99.49%)	
ECMO or LVAD, %					
	YES	2 (0.53%)	10 (0.72%)	12 (0.68%)	0.685
	NO	376 (99.47%)	1374 (99.27%)	1750 (99.32%)	
Diaphragmatic paralysis, %					
	YES	2 (0.53%)	13 (0.94%)	15 (0.85%)	0.442
	NO	376 (99.47%)	1371 (99.06%)	1747 (99.15%)	
Hypoxemia, %					
	YES	20 (5.30%)	49 (3.54%)	69 (3.92%)	0.120
	NO	358 (94.70%)	1335 (96.46%)	1693 (96.08%)	
Neurological complications, %					
	YES	0 (0.00%)	2 (0.14%)	2 (0.11%)	0.460
	NO	378 (100%)	1382 (99.86%)	1760 (99.89%)	
HF, %					
	YES	10 (2.65%)	27 (2.0%)	37 (2.10%)	0.404
	NO	368 (97.35%)	1357 (98.0%)	1725 (97.90%)	
MOF, %					
	YES	8 (2.12%)	15 (1.1%)	23 (1.31%)	0.117
	NO	370 (97.88%)	1369 (98.9%)	1739 (98.69%)	
RF, %					
	YES	11 (2.91%)	27 (1.95%)	38 (2.16%)	0.255
	NO	367 (97.09%)	1357 (98.05%)	1724 (97.84%)	
Infection, %					
	YES	12 (3.17%)	27 (1.95%)	39 (2.21%)	0.152
	NO	366 (96.83%)	1357 (98.05%)	1723 (97.79%)	
Outcome, %					
	Cured	375 (99.21%)	1376 (99.42%)	1751 (99.38%)	0.637
	One-month mortality after surgery	3 (0.79%)	8 (0.58%)	11 (0.62%)	

Notes: CPBT: cardiopulmonary bypass time; ACCT: aortic cross-clamp time; MVT: mechanical ventilation time; ECMO: extracorporeal membrane oxygenation; LVAD: left ventricular assist device; HF: hepatic failure; MOF: multiple organ failure; RF: renal failure.
